# N-Acetylcysteine in Neurological Disorders: A Systematic Review of Clinical and Translational Evidence Across Seven Disorders

**DOI:** 10.3390/ijms27073076

**Published:** 2026-03-27

**Authors:** Robert Mîndreanu, Irina Camelia Chiș, Alexandra Sevastre-Berghian, Cezar Login, Adina Stan, Teodora Stan, Simona Clichici, Șoimița Suciu

**Affiliations:** 1 Department of Physiology, Iuliu Hațieganu University of Medicine and Pharmacy, 400012 Cluj-Napoca, Romania; 2 Department of Neuroscience, Iuliu Hațieganu University of Medicine and Pharmacy, 400012 Cluj-Napoca, Romania

**Keywords:** N-acetylcysteine, oxidative stress, traumatic brain injury, Parkinson’s disease, Alzheimer’s disease, multiple sclerosis, migraine, amyotrophic lateral sclerosis

## Abstract

N-acetylcysteine (NAC) is a glutathione precursor with established antioxidant and anti-inflammatory properties that has been investigated as a neuroprotective agent across multiple neurological conditions. This systematic review systematically mapped the clinical evidence for NAC across seven neurological disorders. PubMed and Cochrane Library were searched for studies published between 1 January 1995 and 31 December 2025. Twenty-three studies were included: traumatic brain injury (TBI, *n* = 6), Alzheimer’s disease (AD, *n* = 5), Parkinson’s disease (PD, *n* = 5), multiple sclerosis (*n* = 4), amyotrophic lateral sclerosis (*n* = 2), and migraine (*n* = 1); no eligible epilepsy studies were identified. The strongest evidence emerged for acute mild TBI, where early NAC administration significantly improved symptom resolution, and for PD, where combined intravenous/oral NAC improved dopamine transporter binding. In AD, nutraceutical formulations including NAC and other active compounds showed trends toward cognitive stabilization. Most included studies had a high or serious risk of bias, and only eight of 23 assessed oxidative stress biomarkers. NAC demonstrated a favorable safety profile across all conditions. Despite fragmented and heterogeneous evidence, the encouraging signals identified warrant large-scale randomized controlled trials with a standardized biomarker assessment.

## 1. Introduction

The global incidence of neurological disorders follows an upward trend, thus representing a major challenge for healthcare systems [1]. Although there is an important variation in the pathophysiological mechanisms of each neurologic condition, a converging mechanism may be the imbalance between the production of reactive oxygen species (ROS) and the innate antioxidant capacity, a phenomenon which leads to oxidative stress. The central nervous system tends to be vulnerable to oxidative damage caused by intense oxygen consumption and relatively low antioxidant enzymes compared to other tissues. Additionally, the blood–brain barrier impedes the influx of circulatory antioxidants and slows the clearance of byproducts.

In neurological conditions, oxidative stress involves multiple common pathways. Excessive ROS production leads to lipid peroxidation, protein carbonylation and DNA oxidation, which in turn trigger mitochondrial dysfunction and activate pro-apoptotic signaling cascades [2]. In addition, neuroinflammation mechanisms determine the activation of microglia and the release of pro-inflammatory cytokines, thus increasing oxidative stress and creating a cycle of neuronal injury [3].

N-acetylcysteine (NAC) is the stable and bioactive N-acetyl derivative of L-cysteine with a potential neuroprotective effect. Its main mechanism of action involves the provision of cysteine as a substrate of glutathione (GSH) synthesis. By replenishing glutathione stores, NAC helps neutralize the free radicals and protects the cells from oxidative stress damage and apoptosis. Moreover, NAC acts on thiolated proteins by releasing free thiols which may act as antioxidant agents. Direct antioxidant properties of NAC have also been observed in vitro, but the in vivo effect is not significant due to competition with endogenous enzymes [4].

Aside from its role as a GSH precursor, NAC acts as a neuroprotector through multiple molecular mechanisms. NAC modulates the Nrf2/ARE signaling pathway, enhancing the transcription of antioxidant and protective genes. It also attenuates NF-kB mediated neuroinflammation [5]. NAC also demonstrates anti-apoptotic properties through the inhibition of caspase-3 activation [6]. All these mechanisms converge into a potential powerful effect; thus, it is worth investigating NAC across multiple neurological conditions where oxidative stress and neuroinflammation are known pathophysiological pathways.

Although NAC is being used as a mucolytic agent and as an antidote to acetaminophen intoxication [6], its therapeutic potential in neurological disorders has been subject to research in the past few decades. Clinical studies have evaluated the efficacy of NAC in different disorders at different dosing regimens and routes of administration both in acute and chronic settings.

The purpose of this systematic review is to map the breadth and nature of available clinical and translational evidence regarding the efficacy and safety of NAC administration across seven major neurological pathologies—traumatic brain injury (TBI), Alzheimer’s disease (AD), Parkinson’s disease (PD), multiple sclerosis (MS), amyotrophic lateral sclerosis (ALS), migraine, and epilepsy—and identify the gaps in the existing evidence base and outline the potential for future clinical investigation. By including conditions with diverse pathophysiological mechanisms but with shared impact on oxidative stress, this review aims to identify cross-pathological patterns, characterize the existing gaps in the evidence base and identify priorities for future research.

## 2. Methods

### 2.1. Study Design and Protocol

The primary objective was to map the available evidence across neurological conditions rather than to evaluate the treatment effect on a single condition. Also, the marked heterogeneity observed in the preliminary evaluation across the included studies, in terms of study design, intervention protocols, and outcome measurements, impeded meaningful quantitative analysis. The present study was conducted as a systematic review, distinguished by predefined eligibility criteria, formal risk of bias assessment using validated instruments (RoB 2.0 and ROBINS-I), and adherence to PRISMA 2020 guidelines. Narrative synthesis was adopted due to heterogeneity precluding meta-analysis. While a methodological protocol was developed internally prior to the initiation of the study according to the PRISMA 2020 guidelines [7], it was not prospectively registered in an online public registry. The protocol was registered retroactively on the Open Science Framework (registration: DOI: 10.17605/OSF.IO/5DPRG) [8]. The PRISMA 2020 checklist is available in Supplementary Material S1 and the PRISMA 2020 for Abstracts checklist is available in Supplementary Material S2.

### 2.2. Search Strategy

A systematic literature search was conducted in the PubMed and Cochrane Library electronic databases, covering the period between 1 January 1995 and 31 December 2025. The Scopus database was considered during the preliminary phase. A preliminary search was conducted in Scopus using equivalent search strategies, which generated 3414 results across all pathologies. After screening the first 100 records, we determined that the majority of the results were irrelevant to the scope of this review (e.g., preclinical studies, studies addressing other populations than those included in this review). All potentially eligible clinical studies identified were already indexed in PubMed and/or Cochrane Library, with no unique eligible studies being identified in the preliminary search. Given the substantial volume of results, the absence of unique relevant studies in the screened sample, and the limited resources available for screening, Scopus was excluded from the final search strategy. Reference lists of included studies were screened for additional relevant publications. The final database search was executed on 14 January 2026.

The search strategy was done using a combination of MeSH and free key words for intervention (“N-acetylcysteine”, “NAC”) and for the target pathologies (traumatic brain injury, Alzheimer’s disease, Parkinson’s disease, multiple sclerosis, amyotrophic lateral sclerosis, migraine). The complete electronic search strategies, including the exact search strings used for each database and each pathology, are provided in Supplementary Material S3 (Table S1).

The search strategy included specific terms for epilepsy. However, the selection process did not identify clinical studies that fulfilled the predefined eligibility criteria for this disorder.

### 2.3. Eligibility Criteria

The selection criteria followed the PCC (Population, Concept, Context) framework:

Population: Adult or pediatric patients with confirmed diagnosis of: (a) TBI; (b) Alzheimer’s disease; (c) Parkinson’s disease; (d) MS; (e) ALS; (f) migraine; (g) epilepsy. More than 5 patients per study were required. Exclusion criteria included: major comorbidities potentially confounding the effect of NAC (severe hepatic failure, terminal cancer, severe renal failure), epileptic seizures secondary to acute pathologies, and secondary Parkinsonism.
Concept: NAC as monotherapy, adjuvant therapy, or as a component of nutraceutical/antioxidant formulations. Any dosage and any route of administration were accepted. The inclusion of combination therapies was a deliberate methodological choice to capture the full scope of available clinical evidence on NAC in neurological disorders; however, these studies are clearly identified in the results, and the limitations of attributing effects to NAC specifically are discussed. Excluding these studies could have resulted in the omission of a significant body of evidence, thereby limiting the capacity of the systematic review to map the existing literature.
Context: Any clinical setting. Study design: Randomized controlled trials (RCTs), non-randomized interventional studies and observational studies with complete datasets were included. Case reports and case series with 5 or less patients per group were excluded. This threshold was established to balance the need to capture the limited clinical evidence for rare conditions (such as ALS) or understudied disorders while excluding isolated case reports or small case series which are more susceptible to bias and offer limited generalizability.

### 2.4. Study Selection and Data Extraction

**Figure 1:**
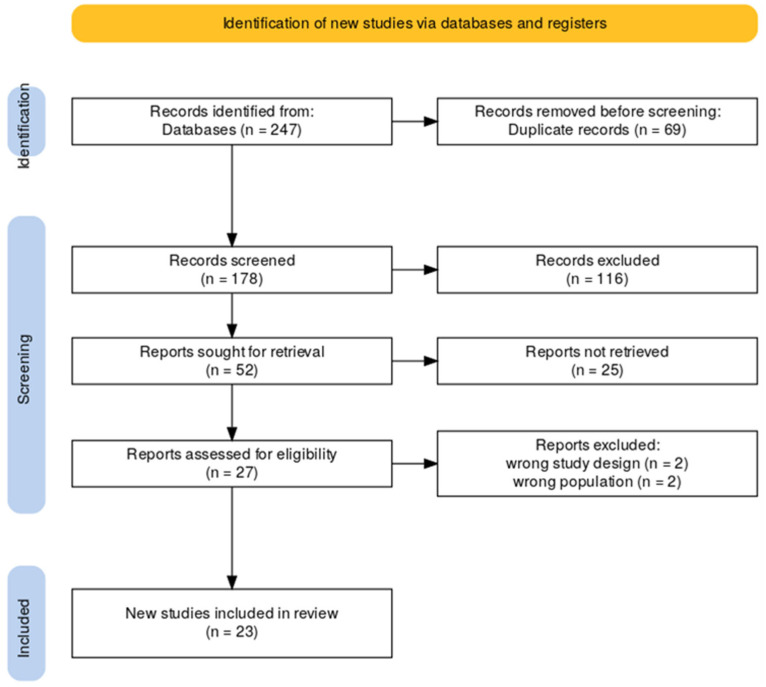
PRISMA flow diagram of the study selection process.

Following the search, all identified citations were exported to a reference manager (Rayyan), and duplicates were removed. A preliminary title and abstract screening were conducted followed by a full-text evaluation and final inclusion. Data extraction was performed by one reviewer. The evaluation was done by two independent investigators, with conflicts resolved by discussion and consensus. The number of search results per pathology and the resulting number of studies included are presented in Table 1. The PRISMA flow diagram detailing the selection process is presented in Figure 1.

For each included study, the following information was extracted:

- Study characteristics: authors, publishing years, country, design.
- Population characteristics: number of participants, age, diagnosis, disease staging.
- Intervention description: NAC dose, route of administration, duration of treatment, co-interventions.
- Comparator characteristics.
- Results: Primary and secondary outcomes, biomarkers, adverse events.

### 2.5. Risk of Bias Assessment

A methodological quality evaluation was conducted for each individual study using the following instruments. Risk of Bias tool version 2 (RoB 2.0) was used for randomized clinical trials and Risk Of Bias In Non-randomized Studies—of Interventions (ROBINS-I) for non-randomized studies. The risk of bias assessment was performed by one reviewer and verified by a second unblinded reviewer. Conflicts were solved by discussion until consensus was reached. Each study received a global evaluation of the risk of bias: for ROBINS-I: low, some concerns, serious or critical; and for RoB 2.0: low risk, some concerns, high risk.

### 2.6. Data Synthesis and Presentation

Results were presented using a narrative synthesis approach, organized by neurological disorder and in the following order of domains: study characteristics, risk of bias assessment, clinical outcomes, biomarker data (where available), adverse effects. A quantitative meta-analysis was considered but deemed not feasible due to the heterogeneity of data. 

## 3. Results

### 3.1. Study Selection

The search identified 115 records from PubMed and 132 records from Cochrane Library. After removing duplicates (69), 178 records were screened and 52 sought for retrieval. Twenty-five reports could not be retrieved due to the following reasons: full-text unavailability (*n* = 3), a lack of access due to paywalls (*n* = 4), clinical trial registrations without initiated status or posted results (*n* = 17), and premature termination of the trial (*n* = 1). Consequently, 27 reports were selected for full-text review. Finally, 23 studies were included: TBI (*n* = 6), AD (*n* = 5), PD (*n* = 5), MS (*n* = 4), ALS (*n* = 2), migraine (*n* = 1). No eligible study regarding epilepsy was found.

### 3.2. Overview of Evidence

Table 2 provides an overview of the evidence. The 23 studies span 1995 to 2025, and the study designs were: 12 RCTs, 1 phase I RCT, five open-label single-arm, three non-randomized comparative, one non-randomized multicomponent, and one metabolomics analysis on an existing cohort. Sample sizes ranged from six to 110 and NAC doses from 600 to 6000 mg/day orally, 50 mg/kg IV. Treatment duration ranged between 7 days and 12 months. According to the results of the study, an evidence gap map has been produced (Table 3).

### 3.3. Risk of Bias Assessment

The risk of bias evaluation has been achieved using the Revised Cochrane Risk of Bias tool for randomized trials (RoB 2.0) and Risk Of Bias In Non-randomized Studies—of Interventions (ROBINS-I) for the non-randomized study and is presented in Table 4.

### 3.4. NAC in Traumatic Brain Injury

Traumatic brain injury (TBI) represents a major cause of death and disability worldwide, affecting approximately 69 million people annually [31]. The pathophysiology of TBI implies a primary mechanical injury followed by a cascade of secondary injuries including oxidative stress, neuroinflammation, mitochondrial dysfunction, excitotoxicity and disturbance of the blood–brain barrier [32]. Oxidative stress plays a role in the secondary cerebral insult following TBI, with an increased production of ROS and reactive nitrogen species, depletion of endogenous antioxidants such as GSH and accumulation of lipid peroxidation byproducts [33]. NAC, as a precursor to cysteine and GSH, with direct antioxidant and anti-inflammatory properties, represents a promising therapeutic candidate for TBI.

The systematic search in PubMed and Cochrane Library databases identified six clinical studies that evaluated NAC in patients with TBI.

#### 3.4.1. Clinical and Neuroimaging Outcomes

Hoffer et al. [8] conducted an RCT on the use of NAC in mild TBI in active military personnel in Iraq. The primary objective showed that treatment with NAC was significantly superior to placebo for the resolution of symptoms at day 7 (OR = 3.6, *p* = 0.006). A time-to-treatment effect was observed, as subjects who were given NAC in the first 24 h from the blast had an 86% chance of complete symptom resolution versus 42% in the early placebo group (*p* < 0.01). Individual symptom improvement was observed in dizziness, hearing loss, headache, impaired memory, sleep disturbance and neurocognitive dysfunction. There was no significant improvement between the treated and placebo group in those where the treatment was initiated >24 h post-blast, suggesting a therapeutic window.

Gouda et al. [9] evaluated high-dose oral or enteral NAC in patients with moderate-to-severe TBI. The Glasgow Coma Scale showed significant improvement in the NAC group from the baseline at day 7 (*p* = 0.001) compared to control. The length of stay in the intensive care unit was significantly shorter in the NAC group (*p* = 0.038). There was no difference in mortality between groups.

Clark et al. [11] conducted a pharmacokinetic phase I trial with NAC and probenecid in children with severe TBI. The primary endpoint was the detectable concentration of NAC in cerebrospinal fluid (CSF) (269–468 ng/mL at 24–72 h). No significant differences were found in intracranial pressure, temperature or intracranial pressure-directed therapies. The Glasgow Outcome Scale at 3 months showed no significant difference between groups.

Vedaei et al. [10] evaluated NAC in patients with chronic symptoms of mild TBI (defined as more than 6 months post-lesion). Neuroimaging revealed a significant group-by-time effect on the resting-state functional MRI in the default mode network, the sensory–motor network and emotional circuits. Cognitive performance improvements were correlated with resting-state fMRI over the 3 months of treatment duration (*p* < 0.05). Neuropsychological tests showed a tendency towards improvement in anxiety, depression and post-concussion symptoms.

Amen et al. [13] used NAC as part of a multicomponent intervention in retired American Football players. There was significant improvement in general cognitive function (*p* < 0.001), processing speed (*p* = 0.026), attention (*p* = 0.025), reasoning (*p* = 0.006), and memory (*p* = 0.022). Brain SPECT imaging showed significant increases in cerebral blood flow in the prefrontal cortex, anterior cingulate, parietal and occipital lobes and the cerebellum (*p* < 0.001). Self-reported improvements included memory (69%), attention (53%), disposition (38%), motivation (38%), and sleep (25%).

#### 3.4.2. Biomarkers

Gouda et al. [9] reported that malondialdehyde (MDA) was significantly decreased in the NAC group (*p* < 0.001). Interleukin-6 was significantly decreased in the NAC group (*p* < 0.001). Neuron-specific enolase was significantly decreased in the NAC group (*p* < 0.001). Protein S100B was significantly decreased in the NAC-treated group (*p* = 0.003).

Hagos et al. [12] conducted a neuropharmacometabolomic analysis of the CSF from the pediatric TBI study. Seven glutathione-centered pathways were enriched in the CSF of TBI patients treated with NAC + probenecid versus placebo. Two glutathione-centered metabolic networks were identified. Distinct metabolomic signatures differentiated TBI control, treatment and placebo groups.

#### 3.4.3. Adverse Effects

NAC had a favorable safety profile in all the included studies on TBI. Hoffer et al. [8] reported no adverse effects with oral NAC 4 g/day for 4 days followed by 3 g/day in blast-injured soldiers. Gouda et al. [9] reported no adverse effects attributable to the treatment with high-dose oral or enteral NAC in moderate-to-severe TBI. Clark et al. [11] reported no adverse effects in the treatment group, while a patient in the placebo group withdrew due to a skin rash. Vedaei et al. [10] found that NAC was well tolerated without major adverse effects over the 3-month treatment period. Amen et al. [13] reported no significant adverse effects when using the complex formulation supplement containing NAC.

### 3.5. NAC in Alzheimer’s Disease

AD is a chronic neurodegenerative disorder characterized by progressive cognitive decline, behavioral disturbances and memory deficits. Oxidative stress plays an important role in its pathogenesis, with evidence suggesting an increase in lipid peroxidation, protein oxidation and DNA deterioration in affected cerebral tissues. Moreover, low GSH levels have been observed in AD patients, oxidative stress being observed even before the clinical diagnosis [34]. NAC has a potential therapeutic role in AD mainly due to its antioxidant properties, counterbalancing the GSH deficit and the oxidative stress [35].

Following the systematic search, according to the protocol, five studies concerning the effects of NAC in AD were identified: one assessing NAC monotherapy and four assessing nutraceutical formulations (NFs).

#### 3.5.1. Clinical and Neuroimaging Outcomes

Regarding the reported effects on cognitive test results, Adair et al. [14] found no significant difference in primary outcome measures (Mini-Mental State Examination [MMSE] or Activities of Daily Living [ADL]) at 24 weeks. A non-significant trend toward improvement was observed in MMSE at 12 weeks (*p* = 0.056). However, on secondary measures, significant improvements were found in letter fluency (*p* = 0.008) and Wechsler Memory Scale (WMS) immediate figure recall (*p* = 0.011) at 3 months. A composite cognitive measure saw improvement with NAC usage both at midpoint (*p* = 0.028) and final evaluation (*p* = 0.004). Chan et al. [15] reported that participants significantly improved on the Dementia Rating Scale (DRS) at 6 months (*p* < 0.02) and showed clinical significance in the Clock Drawing Test (CLOX-1 and CLOX-2) over the 12-month period, without attaining statistical significance (*p* < 0.18 at 12 months). Effect sizes greater than 0.8 were achieved for the DRS at 6 months. Remington et al. [16] found that NFs delayed cognitive decline on the DRS for approximately 6 months compared to placebo, and CLOX-1 showed a 20% decline in the NF group versus a 72% decline in the placebo group at 3 months. Remington et al. [17] reported significant improvement in the NF group versus placebo within 3 months on CLOX-1 (*p* = 0.0083, 95%CI [0.448, 2.93]) and the DRS (*p* = 0.0266, 95%CI [0.17, 2.72]). Remington et al. [18] found that participants maintained baseline cognitive performance over 12 months, contrasting with typical decline observed in historical placebo groups.

Chan et al. [15] reported that caregivers observed improvement in multiple Neuropsychiatric Inventory (NPI) domains, with the greatest effects on irritability and agitation/aggression. Compared to historical placebos, the performance was equivalent to donepezil and exceeded galantamine and their respective placebos. Remington et al. [16] reported approximately 30% improvement in NPI by institutional caregivers over 9 months. Remington et al. [17] found that, during the randomized phase, no significant improvements in total NPI were observed. However, during open-label extensions, caregivers reported statistically significant improvement in NPI (*p* = 0.0204 for the 3-month cohort at 6 months). Remington et al. [18] found that participants maintained the baseline BPSD at 12 months.

Adair et al. [14] reported no significant change in ADL scale. Chan et al. [15] showed a maintenance of ADCS-ADL performance for >28 months. Remington et al. [16] reported improvement in ADCS-ADL at 6 and 9 months. Remington et al. [17] found no significant change in ADL for either cohort. Remington et al. [18] reported no significant change in ADL over 12 months.

#### 3.5.2. Biomarkers

Adair et al. [14] measured peripheral blood markers of oxidant stress (superoxide dismutase, glutathione peroxidase activity, glutathione levels, and thiobarbiturate-reactive substances). No significant differences were found between treatment groups’ biomarkers.

#### 3.5.3. Adverse Effects

In general, NAC was well tolerated in the studies. Adair et al. [14] reported that treatment was well tolerated and no subjects discontinued due to adverse effects. Reported symptoms included fatigue (26% vs. 17% placebo), headache (26% vs. 6%), appetite change (22% vs. 6%), and disposition change (22% vs. 11%), with no significant difference between groups for any adverse effect. Chan et al. [15] reported no serious adverse events, and the NF was well tolerated throughout the 12-month study and 16-month extension. Remington et al. [16] reported no serious adverse events, with one participant refusing medications, though this was not attributed to the NF. Remington et al. [17] reported no serious adverse events throughout the study. Remington et al. [18] reported no serious adverse events over 12 months.

### 3.6. NAC in Parkinson’s Disease

Parkinson’s disease is a chronic neurodegenerative disorder characterized by the loss of dopaminergic neurons in substantia nigra. Oxidative stress plays an essential role in its pathogenesis, with evidence of a decrease in nigral glutathione and increase in ROS during the disease’s progression [36]. NAC, as a precursor to cysteine, represents a potential therapeutic option to counteract the glutathione deficiency and oxidative stress in PD.

Our systematic search in PubMed and Cochrane databases has identified five clinical studies corresponding to the inclusion and exclusion criteria.

#### 3.6.1. Clinical and Neuroimaging Outcomes

Two studies reported significant improvement in the Unified Parkinson’s Disease Rating Scale (UPDRS) score in NAC-treated groups. Monti et al. [19] found an average improvement of 12.9% in the UPDRS total score in the NAC group (*p* = 0.01) compared to the lack of significant change in the control group. Monti et al. [20] reported significant improvement of PD symptoms in the NAC group vs the control group (*p* = 0.0001), and the change in the UPDRS score was significantly correlated with the change in DATscan. Yulug et al. [26] found no significant improvement in motor function in the treatment group vs. placebo.

Yulug et al. [23] demonstrated a significant improvement in cognitive function in the treated group. Montreal Cognitive Assessment (MoCA) scores improved significantly (*p* < 0.001) after 84 days of treatment, compared to the lack of improvement in the placebo group (*p* = 0.05). In patients with severe PD (low MoCA scores), the combined metabolic activators (CMA) group was associated with a significant decrease in cognitive decline (*p* = 0.001).

Yulug et al. [23] evaluated the cerebral connectivity changes using functional MRI in resting state and observed a significant increase in the anterior salience network activity in the CMA-treated group, but not in the placebo group. This change was correlated with an increase in cognitive function, suggesting that metabolic stimulation may influence the cerebral networks involved in cognition.

Two studies have evaluated the effects of NAC on the dopamine transporter (DAT) binding using DaTscan SPECT imaging. Monti et al. [19] found a significant increase in DAT binding in the caudate nucleus (average increase 4.4–7.8%, *p* < 0.05) and putamen (*p* < 0.05) in the NAC group, while the control group did not show significant changes. Monti et al. [20] reported a significant increase in DAT binding in the caudate nucleus and putamen (average increase 3.4–8.3%, *p* < 0.05) in the NAC group, and DaTscan images confirmed visually apparent improvement in post-treatment DAT binding.

#### 3.6.2. Biomarkers

Holmay et al. [21] showed that intravenous (IV) administration of NAC increased cerebral GSH concentrations, measured by 7 Tesla magnetic resonance spectroscopy (MRS). The maximum mean increases in cerebral GSH in the occipital cortex reached 55% in patients with PD. Cerebral GSH peaked at approximately 90–110 min after the start of the NAC infusion, and the subjects with the highest percentual change in blood GSH had also the highest change in brain GSH. Coles et al. [22] evaluated the effect of repeated oral NAC doses (6000 mg/day for 4 weeks) and found that although the measurements of blood antioxidants (catalase, GSH/GSSG) increased significantly from the initial value, no significant increase in brain GSH was detected. This fact may be tied to the low oral bioavailability of NAC.

Holmay et al. [21] found that the GSH/GSSG ratio increased after the NAC perfusion, peaking at 60–75 min, and the increase was observed in all PD patients. Coles et al. [22] reported that after 28 days of oral NAC administration, a significant increase in the catalase and GSH/GSSG ratio was observed in comparison with the initial value, but the lipid peroxidation markers (4-hydroxynonenal [4-HNE] and MDA) were unchanged.

#### 3.6.3. Adverse Effects

In general, NAC was well tolerated in the analyzed studies. Monti et al. [19] reported no significant adverse effects, and no patient discontinued the study. Monti et al. [20] found that treatment was generally well tolerated, with no significant adverse effects reported. Coles et al. [22] noted that three out of five patients with PD reported adverse effects at high oral doses (6000 mg/day), with two adverse effects attributed to the study medication, abdominal pain and constipation, both of which resolved after treatment discontinuation. Yulug et al. [23] reported that CMA treatment was well tolerated, with no significant differences between the adverse effects between the CMA and placebo groups.

### 3.7. NAC in Multiple Sclerosis

Multiple sclerosis (MS) is a chronic autoimmune neurodegenerative disorder of the central nervous system with an estimated 2.8 million people worldwide suffering from the disease [37]. From a pathophysiological perspective it is characterized by demyelination and axonal damage which lead to progressive neurological disability. Oxidative stress plays an important role in the evolution of MS, with evidence of increased ROS production and depletion of endogenous antioxidants such as glutathione in both brain tissue and blood samples [38]. In MS patients compared to healthy controls, lower brain GSH levels were found and a decline in frontal GSH in progressive MS was associated with clinical worsening [39].

The systematic search in PubMed and Cochrane Library databases retrieved four clinical studies evaluating the use of NAC in patients with MS.

#### 3.7.1. Clinical and Neuroimaging Outcomes

Krysko et al. [26] conducted a double-blind RCT on the use of NAC for progressive MS-related fatigue. The Modified Fatigue Impact Scale improved in both groups in week 4, but the NAC group showed a mean improvement of 11 points vs. 18-point improvement in the placebo group (*p* = 0.33, not significant). In week 6 (2 weeks off treatment), more sustained improvement was reported in the NAC group, though not statistically significant. A strong placebo effect was observed, and no significant effect of oral NAC three times a day on fatigue in progressive MS was observed in this small pilot study.

Khalatbari Mohseni et al. [27] evaluated the effects of NAC on neuropsychiatric symptoms in relapsing–remitting multiple sclerosis. Hospital Anxiety and Depression Scale (HADS)—Anxiety (HADS-A) scores decreased significantly in the NAC group compared to placebo (−1.6 ± 2.67 vs. 0.33 ± 2.83; *p* = 0.02). Intra-group analysis showed that NAC supplementation significantly reduced HADS-A scores at 8 weeks (10.05 ± 1.83 to 8.38 ± 2.56; *p* = 0.01). HADS–Depression scores showed no significant differences between groups (*p* = 0.05).

Monti et al. [25] evaluated the effect of NAC on cerebral glucose metabolism using FDG PET/MRI. A significant increase in cerebral glucose metabolism was found in the NAC group compared to control in some brain regions, including the caudate nucleus, inferior frontal gyrus, lateral temporal gyrus, and middle temporal gyrus (*p* < 0.05). Self-reported assessments on cognition improved significantly in the NAC group compared to control (*p* < 0.05), and self-reported assessments on attention also improved significantly in the NAC group compared to control (*p* < 0.05).

Schipper et al. [24] assessed MRI parameters during the combined therapy with NAC and glatiramer acetate. No significant effect on the Gadolinium-enhancing lesions or on the cerebral volume over 36 weeks of treatment was found. The average number of new T2 lesions was 1.43 at week 0, 1.33 at week 32, and 0.17 at week 36, though this was not statistically significant. Only one relapse occurred among six patients during the 36 weeks of combined treatment, compared to 12 relapses in the year prior to entering the study.

#### 3.7.2. Biomarkers

Khalatbari Mohseni et al. [27] reported that serum MDA decreased significantly in the NAC group compared to placebo (−0.33 [−5.85 to 2.50] vs. 2.75 [−0.25 to 5.22] μmol/L; *p* = 0.03). Serum nitric oxide (NO) and erythrocyte glutathione (GSH) saw no significant change between groups. Schipper et al. [24] reported that the erythrocyte GSSG/GSH ratio had a decreasing trend in the NAC group, although it was not statistically significant (0.082 → 0.065 → 0.061 at 0, 16, 36 weeks; *p* = 0.09). There were no significant changes in plasma protein carbonyls, 8-epiPGF2α, 8-hydroxy-2-deoxyguanosine, tocopherols, or carotenoids.

Krysko et al. [26] used a 7T MR spectroscopy to measure brain GSH. No significant differences between NAC and placebo groups in brain GSH levels were found. However, GSH/total creatine concentrations were higher in those where the MR spectroscopy was performed closer in time to the NAC dose, suggesting a potential transient effect.

#### 3.7.3. Adverse Effects

NAC demonstrated a good safety profile in all MS studies. Schipper et al. [24] found that NAC 5 g/day for 36 weeks was safe and well tolerated, with the most frequently observed adverse effects being headache (*n* = 3), abdominal distension (*n* = 3), and asthenia (*n* = 2). Monti et al. [25] reported that NAC IV and orally for 2 months was well tolerated with no specific adverse effects reported. Krysko et al. [26] reported at least one adverse effect in 60% of the NAC group vs. 80% of the placebo group (*p* = 0.75) with a 94% adherence rate. Khalatbari Mohseni et al. [27] reported a good tolerance for NAC 1200 mg/day for 8 weeks, mentioning one patient who was excluded due to shortness of breath, which resolved on discontinuation. No gastrointestinal or major side effects were reported in the remaining participants.

### 3.8. NAC in Amyotrophic Lateral Sclerosis

ALS is a fatal and incurable neurodegenerative disorder, characterized by the degeneration of upper and lower motor neurons. Its prevalence ranges between 1.57 per 100,000 and 11.8 per 100,000 [40], with a median survival from onset ranging from 20 to 48 months [41].

The involvement of free radicals in the etiopathogenesis of ALS received significant validation with the discovery of the mutated SOD1 gene associated with the familial form of ALS [42].

Following the systematic search in PubMed and Cochrane Library, two studies have been found evaluating the effectiveness of NAC in patients with ALS: a randomized controlled trial of NAC in monotherapy and an open-label study with a combination of antioxidants.

#### 3.8.1. Clinical and Neuroimaging Outcomes

Louwerse et al. [28] conducted a double-blind placebo-controlled RCT evaluating subcutaneous NAC (50 mg/kg/day) for 12 months in 110 ALS patients (54 NAC, 56 placebo). Regarding the primary outcome of survival, at the end of the 12-month study period, survival was higher in the NAC group (35/54, 65%) compared to placebo (30/56, 54%), but the difference did not reach statistical significance (*p* = 0.31; hazard ratio 0.74, 95% CI: 0.41–1.33). A subgroup analysis by onset type showed a trend toward a benefit in patients with limb onset (74% vs. 51%, HR 0.5, *p* = 0.06), while patients with bulbar onset showed no benefit (44% vs. 62%, HR 1.66, *p* = 0.36). Concerning secondary outcomes of disease progression, no significant differences were observed between groups for muscle strength measured by the Medical Research Council (MRC) score (*p* = 0.34), forced vital capacity (FVC; *p* = 0.40), or Activities of Daily Living assessed by the Barthel index (*p* = 0.06). Notably, bulbar function deteriorated more rapidly in the NAC group (*p* < 0.01), which the authors attributed to a possible mucolytic effect of NAC increasing the secretion burden in patients with impaired swallowing.

Vyth et al. [29] conducted an open-label study comparing 36 ALS patients treated with sequential antioxidant therapy against 107 historic controls. The treatment protocol included NAC and dithiothreitol/dithioerythritol (DTT/DTE) administered to all 36 patients (100%), vitamins C and E (29/36, 81%), N-acetylmethionine (NAM) (21/36, 58%), and meso-2,3-dimercaptosuccinic acid (DMSA) for patients with metal exposure (7/36, 19%). Median survival was 3.4 years (95% CI: 3.0–4.2) in the treated group compared to 2.8 years (95% CI: 2.2–3.1) in historic controls. The median time from diagnosis to treatment initiation was 8.4 months and the average treatment duration was 2.86 years. However, the authors attributed the 6-month survival difference to selection factors (including immortal time bias) rather than to the treatment effect, concluding that “antioxidants neither seem to harm patients with ALS nor seem to prolong survival.”

#### 3.8.2. Biomarkers

No study included systematic assessments of oxidative stress biomarkers or other biological markers. This represents an important limitation, as it does not allow evaluation of the proposed mechanisms of action.

Vyth et al. [29] reported pharmacokinetic data for DTT: it was completely oxidized in serum within 1 min and eliminated from the circulation within 2 h.

#### 3.8.3. Adverse Effects

Louwerse et al. [28]: Fifteen patients prematurely discontinued treatment (12 in the NAC group vs. three in the placebo group). Reasons included insufficient perceived effects (12 patients) and rash with pain at the injection site (three patients). No severe adverse effects were reported.

Vyth et al. [29]: The tolerability profile varied by agent. NAC (subcutaneous) showed good tolerability with local injection-site reactions. DTT/DTE had poor tolerability, with several patients discontinuing due to a loss of appetite, nausea, gastric pain, sweating, facial flushing, and paresthesias. NAM caused less frequent local reactions and was well tolerated. Vitamins C and E and DMSA were very well tolerated with no adverse effects reported. Subcutaneous NAC administration was associated with local reactions including pain, redness, swelling at the injection site, and occasional gastric pain. Three local abscesses were reported but overall tolerability was good.

### 3.9. NAC in Migraine

Migraine is a cerebral neurovascular inflammatory condition, characterized by recurrent episodes of severe headache. During migraine episodes there is an excess of reactive oxygen species contributing to the oxidative stress, especially nitric oxide [43]. Studies have observed an imbalance in oxidative–antioxidant processes in the CSF of patients suffering from migraine. These processes are associated with cortical spreading depression, blood vessel dilatation and trigemino-cervical activation, known steps in the evolution of a migraine attack [44]. NAC, which acts as an antioxidant, could contribute to the production of glutathione, thus representing a potential oxidative stress-reducing treatment in patients with migraine.

The systematic search conducted according to the protocol in PubMed and Cochrane Library has identified only one relevant study which examined the effects of NAC in combination with vitamins C and E in patients suffering from migraine.

#### 3.9.1. Clinical and Neuroimaging Outcomes

Visser et al. [30] reported significant improvements in headache and migraine frequency in the NAC + vitamin C + vitamin E (NEC) treatment group. The mean number of headache episodes per month decreased by 3.0 ± 3.5 in the NEC group (*p* = 0.004) compared to 1.4 ± 2.7 in the sham group (*p* = 0.073), with the between-group difference approaching significance (*p* = 0.052). The mean number of migraine episodes per month decreased significantly more in the NEC group (−1.7 ± 2.9) compared to sham (−0.4 ± 1.8), with a significant between-group difference (*p* = 0.018). The mean number of migraine days per month decreased by 3.1 ± 5.0 in the NEC group (*p* = 0.009), with no significant change in the sham group. The mean number of moderate/severe headache days per month decreased significantly in both groups: NEC (−3.2 ± 4.8, *p* = 0.017) and sham (−1.6 ± 2.5, *p* = 0.016).

The mean total duration of migraines per month decreased significantly in the NEC group (−34.6 ± 54.7 h, *p* = 0.004), but not in the sham group (−9.0 ± 47.6 h, *p* = 0.408). The mean duration of each migraine episode did not change significantly in either group.

The mean monthly headache visual analog scale pain score decreased significantly in the NEC group from 55.5 ± 17.9 mm at baseline to 34.7 ± 30.7 mm in the final month (*p* = 0.013), representing a reduction of 20.8 ± 31.1 mm. No significant change was observed in the sham group (−6.9 ± 21.8 mm, *p* = 0.379).

The mean number of acute headache medication doses per month decreased significantly more in the NEC group (−5.9 ± 6.3, *p* = 0.002) compared to the sham group (−1.5 ± 3.7, *p* = 0.108), with a significant between-group difference (*p* = 0.007).

#### 3.9.2. Biomarkers

No biomarkers of oxidative stress were measured in this study.

#### 3.9.3. Adverse Effects

NEC was well tolerated in the study. No adverse events were recorded on the self-reporting system during the trial or for up to 6 months following completion. One subject accidentally consumed twice the recommended dose of NEC capsules for 1 month without adverse consequences, and blood tests were normal on review [30].

### 3.10. NAC in Epilepsy

Epilepsy is a chronic neurological condition defined by recurrent unprovoked seizures affecting approximately 50 million people worldwide [45]. Oxidative stress is involved in the pathophysiology of epilepsy, with evidence suggesting that epileptic activity leads to an increased production of reactive oxygen species, mitochondrial dysfunction and a reduction in endogenous antioxidants such as glutathione [46]. Considering the NAC’s role as a precursor to glutathione and its direct antioxidant properties, it represents a candidate for adjunctive therapy in epilepsy, potentially reducing oxidative damage and seizure frequency.

The systematic search conducted in PubMed and Cochrane Library, using the aforementioned strategy, led to eight results in PubMed and 23 results in Cochrane Library. Following the title and abstract screening and the full text review using the inclusion and exclusion criteria, no studies met the eligibility criteria for this systematic review.

The absence of clinical studies underlines a significant void in the current literature regarding the use of N-acetylcysteine in epilepsy. This finding underscores the need for randomized controlled trials to evaluate the efficacy and safety of NAC as adjunctive therapy in patients with epilepsy.

## 4. Discussion

### 4.1. Summary of Evidence

This systematic review maps the clinical evidence from 23 studies that evaluated NAC in seven major neurological disorders, presenting a fragmented landscape of current clinical research. The distribution of evidence was unequal: TBI was the most studied disorder (six studies), followed by PD and AD (five studies each), MS (four studies), while ALS (two studies), Migraine (one study) and Epilepsy (zero studies) were underrepresented. The study design varied from double-blind placebo-controlled RCT to open-label one arm trials and the sample size varied significantly, thus reflecting the scarcity of reliable research evidence regarding NAC in neurology.

The strongest clinical evidence came from three main domains. In mild-TBI, the RCT done by Hoffer et al. [8] demonstrated that NAC administration in the first 24 h from the injury led to a complete resolution of symptoms in 86% of subjects by day 7. These results align with the findings of Gouda et al. [9], who reported significant improvement in Glasgow Coma Scale and a reduction in the length of stay in the intensive care unit in patients with moderate–severe TBI in whom high-dose IV NAC was administered. In Parkinson’s disease, two independent studies (Monti et al. [19,20]) demonstrated consistent improvement in the dopamine transporter binding, accompanied by improvement in the UPDRS motor score. In Alzheimer’s disease, a series of studies done by Remington et al. [16,17,18] showed that nutraceutical formulations containing NAC slowed the cognitive decline over 3 to 12 months. However, as these formulations included multiple bioactive compounds, the observed effects cannot be attributed specifically to NAC.

An important cross-pathological pattern appeared in regard to the route of administration. IV and combined IV/oral protocols demonstrated more robust effects rather than oral administration. This fact was most clearly stated in the study by Holmay et al. [21] on PD where they reported an increase of 55% in cerebral glutathione after a single dose of IV NAC, while in the Coles et al. [22] study there was no significant increase in cerebral GSH after four weeks of high-dose oral NAC. Similarly, in TBI, IV protocols (Gouda et al. [9]); Vedaei et al. [10]) showed clearer changes in biomarkers when compared to exclusively oral administration. This pattern raises significant questions regarding the oral bioavailability of NAC and suggests that the designs of future clinical studies should consider the route of administration as a critical variable.

The methodological quality of the included studies was generally low: seven studies were rated as having a high risk of bias, seven as serious, and one as critical, while none achieved a low risk rating. The most common sources of bias were small sample sizes, open-label designs without placebo control, high attrition rates and the absence of registered protocols. These limitations substantially reduce confidence in the findings across all conditions and should be taken into account when assessing the clinical validity of the information presented in this review.

### 4.2. Molecular Mechanisms and Biomarker Evidence

An important inquiry is if the clinical effects of NAC can be correlated with the proposed molecular mechanisms. NAC is firstly a prodrug which supplies cysteine used in glutathione synthesis, thereby replenishing the antioxidant that constitutes the main intracellular defense against oxidative stress. Beyond this role, NAC modulates the Nrf2/ARE signaling pathway, amplifying the transcription of detoxification enzymes and antioxidant genes. NAC also attenuates NF-κB-mediated neuroinflammation and inhibits caspase-3-dependent apoptosis [4,5,6]. All these mechanisms finally converge on an oxidative stress–neuroinflammation–apoptosis axis which is common ground to all the neurological disorders reviewed in the present work.

However, the biomarker evidence derived from the included studies provided only partial support for the aforementioned mechanisms, mainly due to the lack of measurements in most studies. Only eight out of the 23 studies measured biomarkers of oxidative stress, representing a major deficit in objective measurements of NAC’s effects. A systematic summary of all biomarker measurements, including the direction of effect, is provided in Supplementary Material S4, Table S2. The most robust biomarker evidence came from the study by Holmay et al. [21] into PD where they demonstrated that IV NAC administration increased brain glutathione concentration, establishing a proof of concept for the GSH precursor mechanism. Coles et al. [22] also showed that the administration of oral NAC increased peripheral antioxidant markers but did not increase the brain GSH levels. This evidence suggests a potential pharmacokinetic impediment caused by the blood–brain barrier in orally administered NAC.

In TBI, Gouda et al. [9] showed a significant reduction in MDA, IL-6, neuron-specific enolase and S100B in the NAC treated group, thus providing evidence that NAC influences oxidative stress and neuroinflammation, with an impact on neuronal injury. The CSF metabolomic analysis conducted by Hagos et al. [12] identified seven glutathione-centered pathways enriched in TBI patients who were treated with NAC and probenecid, thus providing a perspective on the mechanism of action of NAC in acute neurological injury.

Regarding MS, Khalatbari Mohseni et al. [27] reported a significant reduction in serum MDA in patients with NAC supplementation, while Krysko et al. [26] reported that brain GSH concentrations were higher when measurements were done closer in time to the NAC administration, suggesting a potential time-dependent transient pharmacologic effect. No study regarding ALS, AD, or migraine measured intracerebral oxidative biomarkers. Adair et al. [14] measured peripheral oxidative markers in AD but found no significant differences—a finding that may reflect the discrepancy between peripheral and central antioxidant status in neurological disorders rather than a true absence of molecular effect.

The paucity of biomarker evaluation in most included studies represents a critical limitation as it prevents the mechanistic interpretation of clinical findings. There is also a heterogeneity in biomarker selection studies that further complicates inter-pathological comparisons. While some studies measured direct indicators, others utilized downstream markers of oxidative injury or neuroinflammatory mediators. Future studies should use a standardized set of molecular biomarkers that include both markers of glutathione pathway and downstream markers of oxidative injury, allowing for meaningful comparisons across studies and pathologies.

### 4.3. Evidence Gap Map and Critical Research Needs

The evidence gap map produced by the current systematic review (Table 3) identified several critical areas requiring urgent investigation. The most obvious finding was the absence of eligible clinical trials evaluating NAC in epilepsy. This lack of evidence is particularly notable given the well-established role of oxidative stress in seizure generation in epileptogenesis, and preclinical evidence demonstrating that NAC reduces seizure frequency and severity in rodent models [47]. Epilepsy, especially treatment-resistant epilepsy, should be considered as one of the focus points for future research into NAC.

In migraine, the identification of a single-pilot RCT (Visser et al. [30]) that used a combination of NAC and vitamins C and E represents insufficient evidence from which to draw a conclusion, for a condition with an 8–10% prevalence in the general population. The significance of the results (reduction in migraine frequency, duration, acute medication use) requires confirmation in larger monotherapy trials, as the combination therapy design precludes the attribution of effects to NAC specifically. Moreover, the absence of oxidative stress biomarker measurements means that the preclinical rationale linking NAC to migraine pathophysiology remains speculative without clinical mechanistic support. The influence of NAC on the pathophysiology of migraine is compelling: excessive nitric oxide production, cortical spreading depression and trigeminocervical activation involve oxidative stress pathways that could potentially be regulated by glutathione replenishment.

In ALS, both available studies from 1995 and 1996 predate the current understanding of the molecular mechanisms of ALS and the modern outcome measurement tools. The field of ALS therapeutic options has evolved considerably since then, with novel biomarkers (neurofilament light chain, SOD1 activity) that could offer a more accurate evaluation of therapeutic effects. Given the known association between SOD1 mutations, oxidative stress and ALS pathogenesis, modern RCTs evaluating NAC are warranted. 

A methodological challenge identified across the span of our research was the use of NAC as part of nutraceutical formulations. Although the nutraceutical formulation used in AD trials (Remington et al. [16,17,18] and the combined metabolic activator strategy in PD (Yulug et al. [23]) produced some of the most consistent clinical improvements, this practice limits the ability to attribute therapeutic effects specifically to NAC and to evaluate how different doses of NAC would influence the outcome. This limitation applies to 13 of the 23 included studies employing multicomponent formulations, where observed effects cannot be clearly attributed to NAC. Future trial designs should include NAC monotherapy arms to clarify the specific contribution of NAC compared to the potential synergistic or confounding effects of co-administered substances.

### 4.4. Route of Administration and Pharmacokinetic Considerations

The route of administration and dosage varied considerably across the included studies, from 600 mg/day orally to 6000 mg/day orally, and from a 50 mg/kg single intravenous dose to a 150 mg/kg loading dose followed by continuous infusion. This heterogeneity of protocols limits direct comparison possibilities. The oral bioavailability of NAC is estimated at only 6–10% [48]. This is reflected in the findings from the Parkinson’s disease studies: intravenous NAC produced a 55% increase in brain GSH (Holmay et al. [21]) while four weeks of high-dose oral NAC (6000 m g/day) failed to increase brain GSH (Coles et al. [22]). The combined intravenous and oral protocol used by Monti et al. [19,20] in Parkinson’s disease, which produced consistent DaTscan improvements, may represent a pragmatic approach to achieving both sustained peripheral antioxidant effects and acute central effects but is not a feasible approach in current clinical practice.

The concept of a therapeutic window observed in TBI by Hoffer et al. [8] adds another layer of complexity. The finding that NAC’s effect was significantly more potent when administered in a shorter period from the injury suggests that the timing of NAC intervention relative to the acute oxidative injury may be as important as the dose and route of administration.

These findings highlight the need for pharmacokinetic–pharmacodynamic studies to establish dose-dependent responses across different routes of administration. Alternative delivery routes, particularly intranasal administration which bypasses the blood–brain barrier, should be investigated as a non-invasive strategy to improve CNS bioavailability. The absence of a significant increase in brain GSH after oral administration does not exclude therapeutic benefit, as oral NAC may exert indirect neuroprotective effects through the reduction in peripheral oxidative stress and systemic inflammation, as demonstrated by the significant improvements in peripheral biomarkers reported by Coles et al. [22] and Khalatbari Mohseni et al. [27]. Future trials should incorporate both central and peripheral biomarkers to capture the full spectrum of NAC’s pharmacological effects.

Beyond dosing and the route of administration, the included studies exhibited considerable heterogeneity in treatment duration and outcome measures. Even within the same disorder, studies used different clinical scales, biomarkers, and neuroimaging modalities, making direct comparison difficult. This highlights the need for standardized outcome sets and treatment protocols in future NAC trials to enable meaningful cross-study comparisons and eventual meta-analysis.

### 4.5. Comparison with Existing Reviews

Previous reviews examined the therapeutic potential of NAC in neurological and psychiatric conditions, which renders the findings of this present study as confirmatory or novel contributions to the existing literature.

Deepmala et al. (2015) [49] conducted the most comprehensive systematic review of NAC in psychiatry and neurology, with the main focus in psychiatry, identifying favorable evidence across multiple conditions such as autism, addiction disorders, bipolar disorder, depression, schizophrenia, Alzheimer’s disease and mild TBI. Their review, which covered the literature until 2014, identified NAC as a safe intervention with good tolerability, well-established across neuropsychiatric patients. Our findings are consistent with and extend their conclusions in the following ways. First, regarding TBI, the evidence base has grown since 2015, with TBI being classified by Deepmala et al. as having only preliminary evidence, whereas our review now identifies six studies demonstrating both clinical and biomarker efficacy in moderate-to-severe TBI with high-dose IV NAC. Secondly, for Parkinson’s disease, the DaTscan SPECT evidence described in Monti et al. [19,20] provides objective neuroimaging evidence of dopaminergic modulation. Our review also mapped biomarker evidence and identified the gap in oxidative stress measurements across the span of the literature.

Tardiolo et al. (2018) [50] provided a narrative overview of NAC’s effects specifically in neurodegenerative diseases (PD, AD, neuropathic pain, stroke), emphasizing preclinical mechanisms while noting the limited clinical evidence available at that time. Our review complements their work by providing a structured mapping of all available clinical studies in the neurodegenerative conditions they discussed (ALS, AD, PD, MS) as well as conditions not addressed in their work (TBI, epilepsy, migraine). Tardiolo et al. [50] raised the question of NAC’s blood–brain barrier penetrance as a key unresolved issue, whereas our review comes with contrasting evidence from Holmay et al. [21] and Coles et al. [22]’s studies, providing empirical clinical evidence that the route of administration may be a critical determinant of CNS bioavailability.

### 4.6. Limitations

The present systematic review has several methodological limitations that should be considered when interpreting the results.

The study protocol was not prospectively registered in an online public registry such as PROSPERO. Although an internal methodological protocol was developed prior to the initiation of the study, the absence of prospective registration represents a methodological limitation, increasing the potential for reporting bias. This decision was taken primarily due to the exploratory and cross-pathological nature of the review, which does not align with the typical format of PROSPERO registrations that focus on a single well-defined clinical question.

Only two databases (PubMed and Cochrane Library) were searched. Although the Scopus database was evaluated in the preliminary phase, it was excluded because no unique eligible studies beyond those already indexed in PubMed and Cochrane Library were found in the first 100 records. We acknowledge that a full systematic screening of all Scopus results was not performed, and the possibility that some relevant studies may have been missed cannot be entirely excluded. The exclusion of EMBASE and Web of Science may have resulted in omitted studies, particularly those published in non-English journals. Gray literature was not searched, which may lead to a publication bias.

The breadth of covering seven neurological conditions in a single review may limit the detailed analysis for each individual condition. Systematic reviews with meta-analyses for conditions with important evidence bases could provide clearer and more robust conclusions.

Although a risk of bias assessment was performed, the certainty of evidence was not formally evaluated. Most included studies had a high or serious risk of bias, caused by small sample sizes, open-label designs, a lack of placebo controls or high attrition rates. No included study achieved a low risk of bias rating, and the overall evidence strength ratings presented in Table 3 should be interpreted in this context. These limitations significantly reduce confidence in the overall assessment of results and conclusions.

The inclusion of multicomponent formulations including NAC, alongside monotherapy studies, induces complexity in interpretation. In studies employing combination therapies, the observed effect cannot be attributed directly to NAC and the potential for synergistic or antagonistic interactions between co-administered agents cannot be quantified.

Finally, the heterogeneity of interventions (monotherapy versus combination therapy, oral versus intravenous, highly variable doses, durations) and outcome measurement tools across studies impeded a potential quantitative analysis of the data.

### 4.7. Implications for Clinical Practice and Research

NAC does not represent a suitable standard treatment for any of the reviewed conditions. However, with a favorable safety profile, low costs and encouraging research evidence, further investigation is warranted. Research priorities may include: 1. Large-scale RCTs with NAC as monotherapy in neurological disorders, especially where there is a lack of evidence (Epilepsy, Migraine, ALS). 2. Increased use of oxidative stress biomarkers in research. 3. Focused systematic reviews and meta-analyses for PD and TBI. Although methodologically limited, the literature provides strong signals regarding the use of NAC in vivo, justifying future explorations. The utilization of neuroimaging demonstrated the capability of NAC to directly modulate the nervous system in Parkinson’s disease by increasing the binding of DAT, and to increase glucose metabolism in some patients with multiple sclerosis. In TBI, the literature suggests an important therapeutic window, with a superior treatment response when administered in the first 24 h from the acute event. A few studies report a reduction in inflammation and neuronal injury biomarkers when treated with NAC. A recurrent subject is the safety profile of NAC, favorable in all studied disorders. A systematic summary of adverse events across all included studies is provided in Supplementary Material S5, Table S3. Most adverse effects were light, including local reactions with parenteral administration or gastrointestinal symptoms with high-dose oral administration.

## 5. Conclusions

This systematic review mapped clinical evidence across seven neurological disorders (with no eligible epilepsy studies), resulting in 23 studies revealing fragmented and heterogeneous evidence but encouraging potential. NAC shows significant neuroprotective effects in acute mild TBI (symptom resolution with NAC monotherapy), PD (DaTscan improvement with combined IV/oral NAC) and AD (cognitive stabilization with nutraceutical formulations containing NAC, though the specific contribution of NAC cannot be isolated from co-administered compounds). However, the overall quality of evidence remains low to moderate, with most studies being exploratory and limited by small sample sizes and a high risk of bias. These findings should be considered preliminary and require confirmation through adequately powered randomized controlled trials. There are significant gaps in knowledge, especially for epilepsy, migraine and ALS where there is a scarcity or lack of evidence. Large-scale RCTs with molecular biomarker assessment are warranted.

## Figures and Tables

**Table 1:** The number of search results per pathology and studies included in the review.

Database	TBI	AD	PD	MS	ALS	Migraine	Epilepsy	Total
PUBMED	17	27	7	9	8	39	8	115
Cochrane Library	25	40	17	18	7	3	22	132
Included studies	6	5	5	4	2	1	0	23

TBI = traumatic brain injury; AD = Alzheimer’s disease; PD = Parkinson’s disease; MS = multiple sclerosis; ALS = amyotrophic lateral sclerosis.

**Table 2:** Summary of the evidence.

Study	Design	N	Intervention	Duration	Primary Outcome
Traumatic brain injury					
Hoffer et al. [8]	RCT double-blind	81	NAC oral 4 g/day for 4 days followed by 3 g/day	7 days	Symptom resolution at day 7
Gouda et al. [9]	RCT open-label	40	NAC oral or enteral high-dose (150 mg/kg load + 50 mg/kg/4 h)	7 days	Biomarkers, GCS
Vedaei et al. [10]	Longitudinal controlled	50	NAC IV 50 mg/kg/wk + oral 1000 mg/day	3 months	rs-fMRI metrics, cognition
Clark et al. [11]	RCT Phase I double-blind	14	NAC oral + probenecid	72 h	CSF pharmacokinetics
Hagos et al. [12]	RCT metabolomics substudy	12 + 5	NAC oral + probenecid	24 h	CSF metabolomics
Amen et al. [13]	Open-label pragmatic	30	NAC in complex supplement *	6 months	Cognition (MACF), SPECT
Alzheimer’s disease					
Adair et al. [14]	RCT double-blind	43 (23/20)	NAC 50 mg/kg/day oral	6 months	MMSE, ADL
Chan et al. [15]	Open-label pilot	14	NF (folate, B12, vit E, SAM, NAC 600 mg, ALCAR)	12 month + 16 months extension	DRS, CLOX, NPI, ADL
Remington et al. [16]	Placebo-controlled pilot	12 (6/6)	NF (folate, B12, vit E, SAM, NAC 600 mg, ALCAR)	9 months	DRS, CLOX-1, NPI, ADL
Remington et al. [17]	RCT Phase II double-blind multicenter	106 (62/44)	NF (folate, B12, vit E, SAM, NAC 600 mg, ALCAR)	3–6 months + open-label extension	DRS, CLOX-1, NPI, ADL
Remington et al. [18]	Open-label	24	NF (as above)	12 months	DRS, CLOX, NPI, ADL
Parkinson’s disease					
Monti et al. [19]	Pilot RCT	23 (12/11)	NAC IV 50 mg/kg/week + oral 600 mg × 2/day	3 months	DaTscan (DAT binding), UPDRS
Monti et al. [20]	RCT	42 (21/21)	NAC IV 50 mg/kg/week + oral 500 mg × 2/day	3 months	DaTscan (DaT binding), UPDRS
Holmay et al. [21]	Open-label	3 PD + 3 HC	NAC IV 150 mg/kg single dose	2 h	Cerebral GSH (MRS 7T), blood GSH/GSSG
Coles et al. [22]	Open-label	5 PD + 3 HC	NAC 6000 mg/day oral	4 weeks	Cerebral GSH (MRS 3T/7T), UPDRS
Yulug et al. [23]	RCT phase II	64 (32/32)	CMA × 2/day (NAC 2.55 gr/dose + L-serine +NR + L-carnitine tartrate)	84 days	MoCA, MDS-UPDRS, fMRI
Multiple sclerosis					
Schipper et al. [24]	Open-label pilot	7	NAC 5 g/day oral + GA 20 mg/day	36 weeks	Safety, tolerability
Monti et al. [25]	RCT waitlist-controlled	24	NAC IV 50 mg/kg/wk + oral 500 mg × 2/day	2 months	FDG PET glucose metabolism
Krysko et al. [26]	RCT double-blind	15	NAC 1250 mg TID oral	4 weeks	Fatigue (MFIS), safety
Khalatbari Mohseni et al. [27]	RCT double-blind	42	NAC 600 mg × 2/day oral	8 weeks	Oxidative markers, HADS
Amyotrophic lateral sclerosis					
Louwerse et al. [28]	Placebo-controlled double-blind RCT	110 (54 NAC vs. 56 Placebo)	NAC 50 mg/kg/day subcutaneous (monotherapy)	12 months	Survival at 12 months
Vyth et al. 1996 [29]	Open-label with historic controls	143 (36 treated vs. 107 historic controls)	NAC + Vit C/E + NAM + DTT/DTE ± DMSA (combination)	~2.9 years	Survival from diagnosis
Migraine					
Visser et al. [30]	RCT double-blind	35 (19/16)	NEC oral (NAC 600 mg + Vit E 250 IU + Vit C 500 mg) × 2/day	3 months	Headache frequency, migraine days, VAS pain score

NAC = N-acetylcysteine; NAM = N-acetylmethionine; DTT = dithiothreitol; DTE = dithioerythritol; DMSA = meso-2,3-dimercaptosuccinic acid (metal chelator); NF = nutraceutical formulation; SAM = S-adenosyl methionine; ALCAR = acetyl-L-carnitine; DRS = Dementia Rating Scale; MMSE = Mini-Mental State Examination; CLOX = Clock Drawing Test; NPI = Neuropsychiatric Inventory; ADL = Activities of Daily Living; RRMS = relapsing–remitting MS; MS = multiple sclerosis; GA = glatiramer acetate; IV = intravenous; FDG PET = fluorodeoxyglucose positron emission tomography; MFIS = Modified Fatigue Impact Scale; HADS = Hospital Anxiety and Depression Scale; TID = three times daily; DAT = dopamine transporter; UPDRS = Unified Parkinson’s Disease Rating Scale; MRS = magnetic resonance spectroscopy; GSH = glutathione; GSSG = oxidated glutathione; MoCA = Montreal Cognitive Assessment; CMA = combined metabolic activators; NR = nicotinamide riboside; HC = healthy controls; PD = Parkinson’s disease; mTBI = mild traumatic brain injury; GCS = Glasgow Coma Scale; IV = intravenous; CSF = cerebrospinal fluid; rs-fMRI = resting-state functional MRI; MACF = Microcog Assessment of Cognitive Functioning; SPECT = single-photon emission computed tomography. * NAC as part of multicomponent supplement including ginkgo, vinpocetine, alpha-lipoic acid, acetyl-l-carnitine, huperzine A, phosphatidylserine.

**Table 3:** Evidence gap map: NAC in neurological disorders.

Pathology	Total Studies	RCTs	NAC Monotherapy	NAC in Combination	Motor/ Clinical Outcomes	Cognitive Outcomes	Biomarkers of Oxidative Stress	Brain GSH (MRS)	Neuroimaging	Safety Data	Overall Evidence Strength
TBI	6	2	2 (Hoffer et al. [8], Gouda et al. [9])	4	GCS, symptoms (sig. acute mTBI)	Cognitive tests (sig. chronic)	MDA, IL-6, NSE, S100B (sig.)	CSF metabolomics (enriched pathways)	SPECT, rs-fMRI (sig.)	Reported (excellent profile)	Moderate (strongest for acute mTBI)
AD	5	2	1 (Adair et al. [14])	4 (NF studies)	ADL assessed (mixed results)	DRS, MMSE, CLOX NPI (positive trends)	Peripheral markers (1 study, NS)	Not measured	Not performed	Reported (well tolerated)	Low–moderate (combo therapy, same group)
PD	5	3	2 (Monti et al. [19,20])	3	UPDRS (2/3 sig.)	MoCA (sig. with CMA)	Blood GSH, catalase (sig. oral)	7T MRS (IV: +55%; oral: NS)	DaTscan SPECT (sig. 3–8%), rs-fMRI	Reported (well tolerated)	Moderate (consistent DaTscan, but small n)
MS	4	2	1 (Krysko et al. [26])	3	Fatigue (NS), anxiety (sig.), MRI (NS)	Self-reported cognition (sig.)	MDA (sig.), GSSG/GSH (trend)	7T MRS (1 study, NS)	FDG PET (sig.), MRI (NS)	Reported (well tolerated)	Low (small studies, heterogeneous)
ALS	2	1	1 (Louwerse et al. [28])	1 (Vyth et al. [29])	Survival, MRC, FVC, Barthel (no sig. benefit)	Not assessed	Not measured	Not measured	Not performed	Reported (well tolerated)	Very low (dated studies, *n* = 2, since 1996)
Migraine	1	1	None	1 (NEC, Visser et al. [30])	Frequency, VAS, medication use (significant)	Not assessed	Not measured	Not measured	Not performed	Reported (no AEs)	Very low (single study, combo therapy)
Epilepsy	0	0	None	None	No studies	No studies	No studies	No studies	No studies	No studies	No evidence (critical gap)

This evidence gap map summarizes the availability and strength of clinical evidence for NAC across seven neurological disorders. Each column represents a specific evidence domain. Cells are color-coded: green indicates available evidence, yellow indicates limited evidence (1–2 studies or significant methodological concerns), and red indicates no evidence (critical gap). ALS = amyotrophic lateral sclerosis; AD = Alzheimer’s disease; MS = multiple sclerosis; PD = Parkinson’s disease; TBI = traumatic brain injury; RCT = randomized controlled trial; NF = nutraceutical formulation; NEC = NAC + vitamin E + vitamin C; CMA = combined metabolic activators; MRS = magnetic resonance spectroscopy; GSH = glutathione; MDA = malondialdehyde; NS = not significant; sig. = statistically significant; DaTscan = dopamine transporter scan; SPECT = single-photon emission computed tomography; rs-fMRI = resting-state functional MRI; GCS = Glasgow Coma Scale; UPDRS = Unified Parkinson’s Disease Rating Scale; VAS = visual analog scale; AE = adverse event. Overall evidence strength ratings were assigned based on the following criteria: moderate = at least 2 studies including at least one RCT with consistent direction of effect, supported by biomarker or neuroimaging data, despite methodological limitations; low–moderate = multiple studies with consistent trends but significant methodological concerns (e.g., combination therapies, same research group, high risk of bias); low = small number of studies with heterogeneous designs and substantial methodological limitations; very low = one or two studies only, with high risk of bias or dated methodology; no evidence = no eligible studies identified.

**Table 4:** Risk of bias assessment.

Study	Instrument	Overall Assessment (Risk)	Main Concern
Traumatic brain injury			
Hoffer et al. 2013 [8]	RoB 2.0	Some concerns	Partially subjective outcomes, main evaluator was the same for all the patients
Gouda et al. [9]	RoB 2.0	High	Significant attrition, open-label
Vedaei et al. [10]	ROBINS-I	Serious	Non-randomized, no mention of allocation method, no blinding, self-reported clinical measures
Clark et al. [11]	RoB 2.0	Some concerns	Small sample, significant attrition
Hagos et al. [12]	RoB 2.0	Some concerns	Small sample, exploratory study
Amen et al. [13]	ROBINS-I	Critical risk	No control group, multicomponent intervention
Alzheimer’s disease			
Adair et al. [14]	RoB 2.0	Some concerns	Small study without registered protocol
Chan et al. [15]	ROBINS-I	Serious	Lack of control group, open-label, comparison with historic placebo
Remington et al. [16]	RoB 2.0	High	Pilot study with few participants, loss of placebo group
Remington et al. [17]	RoB 2.0	High	Changes in the protocol based on preliminary results
Remington et al. [18]	ROBINS-I	Serious	Open-label, auto-selection, lack of control
Parkinson’s disease			
Monti et al. [19]	RoB 2.0	High	Open-label, lack of placebo
Monti et al. [20]	RoB 2.0	High	Open-label, no blinding, lack of placebo group
Holmay et al. [21]	ROBINS-I	Serious	Small sample size, lack of control group
Coles et al. [22]	ROBINS-I	Serious	Small sample size, lack of control group
Yulug et al. [23]	RoB 2.0	Some concerns	Positive results only from a secondary outcome
**Multiple sclerosis**			
Schipper et al. [24]	ROBINS-I	Serious	Open-label, one arm, small sample size, no randomization
Monti et al. [25]	RoB 2.0	High	Open-label, lack of placebo, lack of blinding
Krysko et al. [26]	RoB 2.0	Some concerns	Small sample size, significant difference between groups’ baseline age
Khalatbari Mohseni et al. [27]	RoB 2.0	Some concerns	High dropout rate
Amyotrophic lateral sclerosis			
Louwerse et al. [28]	RoB 2.0	Some concerns	Mainly due to the differential rate of treatment discontinuation
Vyth et al. [29]	ROBINS-I	Serious	Dominated by the selection bias, immortal time bias, confounding bias
Migraine			
Visser et al. [30]	RoB 2.0	High	Small sample size, massive attrition

RoB 2.0 = Risk of Bias tool version 2; ROBINS-I = Risk Of Bias In Non-randomized Studies—of Interventions.

## Data Availability

No new data were created or analyzed in this study. Data sharing is not applicable to this article.

## Supplementary Materials

The following supporting information can be downloaded at: https://www.mdpi.com/article/10.3390/ijms27073076/s1.

## Abbreviations

The following abbreviations are used in this manuscript:

4-HNE	4-hydroxynonenal
8-epiPGF2α	8-epi-prostaglandin F2α
AD	Alzheimer’s disease
ADCS-ADL	Alzheimer’s Disease Cooperative Study—Activities of Daily Living
ADL	Activities of Daily Living
AE	adverse event
ALCAR	acetyl-L-carnitine
ALS	amyotrophic lateral sclerosis
ARE	antioxidant response element
BPSD	behavioral and psychological symptoms of dementia
CI	confidence interval
CLOX	Clock Drawing Test
CMA	combined metabolic activators
CNS	central nervous system
CSF	cerebrospinal fluid
DAT	dopamine transporter
DaTscan	dopamine transporter scan
DMSA	meso-2,3-dimercaptosuccinic acid
DNA	deoxyribonucleic acid
DRS	Dementia Rating Scale
DTE	dithioerythritol
DTT	dithiothreitol
FDG PET	fluorodeoxyglucose positron emission tomography
fMRI	functional magnetic resonance imaging
FVC	forced vital capacity
GA	glatiramer acetate
GCS	Glasgow Coma Scale
GSH	glutathione
GSSG	oxidized glutathione
HADS	Hospital Anxiety and Depression Scale
HADS-A	Hospital Anxiety and Depression Scale—Anxiety
HC	healthy controls
HR	hazard ratio
IL-6	interleukin-6
IU	International Units
IV	intravenous
MACF	Microcog Assessment of Cognitive Functioning
MDA	malondialdehyde
MDS-UPDRS	Movement Disorder Society—Unified Parkinson’s Disease Rating Scale
MeSH	Medical Subject Headings
MFIS	Modified Fatigue Impact Scale
MMSE	Mini-Mental State Examination
MoCA	Montreal Cognitive Assessment
MRC	Medical Research Council
MRI	magnetic resonance imaging
MRS	magnetic resonance spectroscopy
MS	multiple sclerosis
mTBI	mild traumatic brain injury
NAC	N-acetylcysteine
NAM	N-acetylmethionine
NEC	N-acetylcysteine + vitamin E + vitamin C
NF	nutraceutical formulation
NF-κB	nuclear factor kappa-light-chain-enhancer of activated B cells
NO	nitric oxide
NPI	Neuropsychiatric Inventory
NR	nicotinamide riboside
Nrf2	nuclear factor erythroid 2-related factor 2
NS	not significant
NSE	neuron-specific enolase
OR	odds ratio
PCC	Population, Concept, Context
PD	Parkinson’s disease
PET	positron emission tomography
PRISMA	Preferred Reporting Items for Systematic Reviews and Meta-Analyses
RCT	randomized controlled trial
RoB 2.0	Risk of Bias tool version 2
ROBINS-I	Risk Of Bias In Non-randomized Studies—of Interventions
ROS	reactive oxygen species
RRMS	relapsing–remitting multiple sclerosis
rs-fMRI	resting-state functional magnetic resonance imaging
S100B	S100 calcium-binding protein B
SAM	S-adenosyl methionine
sig.	statistically significant
SOD1	superoxide dismutase 1
SPECT	single-photon emission computed tomography
TBI	traumatic brain injury
TID	three times daily
UPDRS	Unified Parkinson’s Disease Rating Scale
VAS	visual analog scale
WMS	Wechsler Memory Scale

## References

[1] Feigin V.L., Nichols E., Alam T., Bannick M.S., Beghi E., Blake N., Culpepper W.J., Dorsey E.R., Elbaz A., Ellenbogen R.G. (2019). Global, Regional, and National Burden of Neurological Disorders, 1990–2016: A Systematic Analysis for the Global Burden of Disease Study 2016. Lancet Neurol..

[2] Halliwell B. (2006). Oxidative Stress and Neurodegeneration: Where Are We Now?. J. Neurochem..

[3] Mattson M.P., Camandola S. (2001). NF-kappaB in Neuronal Plasticity and Neurodegenerative Disorders. J. Clin. Investig..

[4] Aldini G., Altomare A., Baron G., Vistoli G., Carini M., Borsani L., Sergio F. (2018). N-Acetylcysteine as an Antioxidant and Disulphide Breaking Agent: The Reasons Why. Free Radic. Res..

[5] Oka S., Kamata H., Kamata K., Yagisawa H., Hirata H. (2000). *N*-Acetylcysteine Suppresses TNF-induced NF-κB Activation through Inhibition of IκB Kinases. FEBS Lett..

[6] Samuni Y., Goldstein S., Dean O.M., Berk M. (2013). The Chemistry and Biological Activities of N-Acetylcysteine. Biochim. Biophys. Acta BBA—Gen. Subj..

[7] Page M.J., McKenzie J.E., Bossuyt P.M., Boutron I., Hoffmann T.C., Mulrow C.D., Shamseer L., Tetzlaff J.M., Akl E.A., Brennan S.E. (2021). The PRISMA 2020 Statement: An Updated Guideline for Reporting Systematic Reviews. BMJ.

[8] Hoffer M.E., Balaban C., Slade M.D., Tsao J.W., Hoffer B. (2013). Amelioration of Acute Sequelae of Blast Induced Mild Traumatic Brain Injury by N-Acetyl Cysteine: A Double-Blind, Placebo Controlled Study. PLoS ONE.

[9] Gouda A.R., El-Bassiouny N.A., Salahuddin A., Hamouda E.H., Kassem A.B. (2025). Repurposing of High-Dose N-Acetylcysteine as Anti-Inflammatory, Antioxidant and Neuroprotective Agent in Moderate to Severe Traumatic Brain Injury Patients: A Randomized Controlled Trial. Inflammopharmacology.

[10] Vedaei F., Newberg A.B., Alizadeh M., Zabrecky G., Navarreto E., Hriso C., Wintering N., Mohamed F.B., Monti D. (2024). Treatment Effects of N-Acetyl Cysteine on Resting-State Functional MRI and Cognitive Performance in Patients with Chronic Mild Traumatic Brain Injury: A Longitudinal Study. Front. Neurol..

[11] Clark R.S.B., Empey P.E., Bayır H., Rosario B.L., Poloyac S.M., Kochanek P.M., Nolin T.D., Au A.K., Horvat C.M., Wisniewski S.R. (2017). Phase I Randomized Clinical Trial of N-Acetylcysteine in Combination with an Adjuvant Probenecid for Treatment of Severe Traumatic Brain Injury in Children. PLoS ONE.

[12] Hagos F.T., Empey P.E., Wang P., Ma X., Poloyac S.M., Bayir H., Kochanek P.M., Bell M.J., Clark R.S.B. (2018). Exploratory Application of Neuropharmacometabolomics in Severe Childhood Traumatic Brain Injury*. Crit. Care Med..

[13] Amen D.G., Wu J.C., Taylor D., Willeumier K. (2011). Reversing Brain Damage in Former NFL Players: Implications for Traumatic Brain Injury and Substance Abuse Rehabilitation. J. Psychoact. Drugs.

[14] Adair J.C., Knoefel J.E., Morgan N. (2001). Controlled Trial of N-Acetylcysteine for Patients with Probable Alzheimer’s Disease. Neurology.

[15] Chan A., Paskavitz J., Remington R., Rasmussen S., Shea T.B. (2009). Efficacy of a Vitamin/Nutriceutical Formulation for Early-Stage Alzheimer’s Disease: A 1-Year, Open-Label Pilot Study with an 16-Month Caregiver Extension. Am. J. Alzheimers Dis. Dement..

[16] Remington R., Chan A., Paskavitz J., Shea T.B. (2009). Efficacy of a Vitamin/Nutriceutical Formulation for Moderate-Stage to Later-Stage Alzheimer’s Disease: A Placebo-Controlled Pilot Study. Am. J. Alzheimers Dis. Dement..

[17] Remington R., Bechtel C., Larsen D., Samar A., Doshanjh L., Fishman P., Luo Y., Smyers K., Page R., Morrell C. (2015). A Phase II Randomized Clinical Trial of a Nutritional Formulation for Cognition and Mood in Alzheimer’s Disease. J. Alzheimers Dis..

[18] Remington R., Bechtel C., Larsen D., Samar A., Page R., Morrell C., Shea T.B. (2016). Maintenance of Cognitive Performance and Mood for Individuals with Alzheimer’s Disease Following Consumption of a Nutraceutical Formulation: A One-Year, Open-Label Study. J. Alzheimer’s Dis..

[19] Monti D.A., Zabrecky G., Kremens D., Liang T.-W., Wintering N.A., Cai J., Wei X., Bazzan A.J., Zhong L., Bowen B. (2016). N-Acetyl Cysteine May Support Dopamine Neurons in Parkinson’s Disease: Preliminary Clinical and Cell Line Data. PLoS ONE.

[20] Monti D.A., Zabrecky G., Kremens D., Liang T., Wintering N.A., Bazzan A.J., Zhong L., Bowens B.K., Chervoneva I., Intenzo C. (2019). N-Acetyl Cysteine Is Associated with Dopaminergic Improvement in Parkinson’s Disease. Clin. Pharmacol. Ther..

[21] Holmay M.J., Terpstra M., Coles L.D., Mishra U., Ahlskog M., Öz G., Cloyd J.C., Tuite P.J. (2013). N-Acetylcysteine Boosts Brain and Blood Glutathione in Gaucher and Parkinson Diseases. Clin. Neuropharmacol..

[22] Coles L.D., Tuite P.J., Öz G., Mishra U.R., Kartha R.V., Sullivan K.M., Cloyd J.C., Terpstra M. (2019). Repeated-Dose Oral N-Acetylcysteine in Parkinson Disease: Pharmacokinetics and Effect on Brain Glutathione and Oxidative Stress. Control. Clin. Trial.

[23] Yulug B., Altay O., Li X., Hanoglu L., Cankaya S., Velioglu H.A., Lam S., Yang H., Coskun E., Idil E. (2024). Multi-Omics Characterization of Improved Cognitive Functions in Parkinson’s Disease Patients After the Combined Metabolic Activator Treatment: A Randomized, Double-Blinded, Placebo-Controlled Phase II Trial. Brain Commun..

[24] Schipper H.M., Arnold D., Grand’Maison F., Melmed C., Moore F., Levental M., Su H., Constantin M., Stril J.-L., Godin J. (2015). Tolerability and Safety of Combined Glatiramer Acetate and N-Acetylcysteine in Relapsing-Remitting Multiple Sclerosis. Clin. Neuropharmacol..

[25] Monti D.A., Zabrecky G., Leist T.P., Wintering N., Bazzan A.J., Zhan T., Newberg A.B. (2020). N-Acetyl Cysteine Administration Is Associated with Increased Cerebral Glucose Metabolism in Patients with Multiple Sclerosis: An Exploratory Study. Front. Neurol..

[26] Krysko K.M., Bischof A., Nourbakhsh B., Henry R.G., Revirajan N., Manguinao M., Nguyen K., Akula A., Li Y., Waubant E. (2021). A Pilot Study of Oxidative Pathways in MS Fatigue: Randomized Trial of N-acetyl Cysteine. Ann. Clin. Transl. Neurol..

[27] Mohseni G.K., Hosseini S.A., Majdinasab N., Cheraghian B. (2023). Effects of N-acetylcysteine on Oxidative Stress Biomarkers, Depression, and Anxiety Symptoms in Patients with Multiple Sclerosis. Neuropsychopharmacol. Rep..

[28] Louwerse E.S., Weverling G.J., Bossuyt P.M.M., Meyjes F.E.P., De Jong J.M.B.V. (1995). Randomized, Double-Blind, Controlled Trial of Acetylcysteine in Amyotrophic Lateral Sclerosis. Arch. Neurol..

[29] Vyth A., Timmer J.G., Bossuyt P.M.M., Louwerse E.S., De Jong J.M.B.V. (1996). Survival in Patients with Amyotrophic Lateral Sclerosis, Treated with an Array of Antioxidants. J. Neurol. Sci..

[30] Visser E.J., Drummond P.D., Lee-Visser J.L.A. (2020). Reduction in Migraine and Headache Frequency and Intensity with Combined Antioxidant Prophylaxis (*N*-acetylcysteine, Vitamin E, and Vitamin C): A Randomized Sham-Controlled Pilot Study. Pain Pract..

[31] Dewan M.C., Rattani A., Gupta S., Baticulon R.E., Hung Y.-C., Punchak M., Agrawal A., Adeleye A.O., Shrime M.G., Rubiano A.M. (2019). Estimating the Global Incidence of Traumatic Brain Injury. J. Neurosurg..

[32] Freire M.A.M., Rocha G.S., Bittencourt L.O., Falcao D., Lima R.R., Cavalcanti J.R.L.P. (2023). Cellular and Molecular Pathophysiology of Traumatic Brain Injury: What Have We Learned So Far?. Biology.

[33] Khatri N., Thakur M., Pareek V., Kumar S., Sharma S., Datusalia A.K. (2018). Oxidative Stress: Major Threat in Traumatic Brain Injury. CNS Neurol. Disord.—Drug Targets.

[34] Roy R.G., Mandal P.K., Maroon J.C. (2023). Oxidative Stress Occurs Prior to Amyloid Aβ Plaque Formation and Tau Phosphorylation in Alzheimer’s Disease: Role of Glutathione and Metal Ions. ACS Chem. Neurosci..

[35] Bavarsad Shahripour R., Harrigan M.R., Alexandrov A.V. (2014). N-Acetylcysteine (NAC) in Neurological Disorders: Mechanisms of Action and Therapeutic Opportunities. Brain Behav..

[36] Perry T.L., Godin D.V., Hansen S. (1982). Parkinson’s Disease: A Disorder Due to Nigral Glutathione Deficiency?. Neurosci. Lett..

[37] Walton C., King R., Rechtman L., Kaye W., Leray E., Marrie R.A., Robertson N., La Rocca N., Uitdehaag B., Van Der Mei I. (2020). Rising Prevalence of Multiple Sclerosis Worldwide: Insights from the Atlas of MS, Third Edition. Mult. Scler. J..

[38] Adamczyk B., Adamczyk-Sowa M. (2016). New Insights into the Role of Oxidative Stress Mechanisms in the Pathophysiology and Treatment of Multiple Sclerosis. Oxid. Med. Cell. Longev..

[39] Choi I.-Y., Lee S.-P., Denney D., Lynch S. (2011). Lower Levels of Glutathione in the Brains of Secondary Progressive Multiple Sclerosis Patients Measured by ^1^H Magnetic Resonance Chemical Shift Imaging at 3 T. Mult. Scler. J..

[40] Wolfson C., Gauvin D.E., Ishola F., Oskoui M. (2023). Global Prevalence and Incidence of Amyotrophic Lateral Sclerosis: A Systematic Review. Neurology.

[41] Chiò A., Logroscino G., Hardiman O., Swingler R., Mitchell D., Beghi E., Traynor B.G., on behalf of the Eurals Consortium (2009). Prognostic Factors in ALS: A Critical Review. Amyotroph. Lateral Scler..

[42] Abati E., Bresolin N., Comi G., Corti S. (2020). Silence Superoxide Dismutase 1 (SOD1): A Promising Therapeutic Target for Amyotrophic Lateral Sclerosis (ALS). Expert Opin. Ther. Targets.

[43] Neri M., Frustaci A., Milic M., Valdiglesias V., Fini M., Bonassi S., Barbanti P. (2015). A Meta-Analysis of Biomarkers Related to Oxidative Stress and Nitric Oxide Pathway in Migraine. Cephalalgia.

[44] Iyengar S., Johnson K.W., Ossipov M.H., Aurora S.K. (2019). CGRP and the Trigeminal System in Migraine. Headache J. Head Face Pain.

[45] Ngugi A.K., Bottomley C., Kleinschmidt I., Sander J.W., Newton C.R. (2010). Estimation of the Burden of Active and Life-time Epilepsy: A Meta-analytic Approach. Epilepsia.

[46] Shin E.-J., Jeong J.H., Chung Y.H., Kim W.-K., Ko K.-H., Bach J.-H., Hong J.-S., Yoneda Y., Kim H.-C. (2011). Role of Oxidative Stress in Epileptic Seizures. Neurochem. Int..

[47] Sharma S., Jain S.K., Gupta P. (2025). Piyush Kumar Therapeutic Potential of N-Acetylcysteine in Epilepsy: A Systematic Review. Neurol. Asia.

[48] Borgström L., Kågedal B., Paulsen O. (1986). Pharmacokinetics of N-Acetylcysteine in Man. Eur. J. Clin. Pharmacol..

[49] Deepmala, Slattery J., Kumar N., Delhey L., Berk M., Dean O., Spielholz C., Frye R. (2015). Clinical Trials of N-Acetylcysteine in Psychiatry and Neurology: A Systematic Review. Neurosci. Biobehav. Rev..

[50] Tardiolo G., Bramanti P., Mazzon E. (2018). Overview on the Effects of N-Acetylcysteine in Neurodegenerative Diseases. Molecules.

