# Supplementary material for: N-Acetylcysteine in Neurological Disorders: A Systematic Review of Clinical and Translational Evidence Across Seven Disorders
Source: Int J Mol Sci. 2026 Mar 27;27(7):3076. doi: 10.3390/ijms27073076 (PMC13074174; doi:10.3390/ijms27073076)
Supplement: Supplementary file 1 [file ijms-27-03076-s001.zip › Supplementary material S3.pdf]

Table S1. Complete electronic search strategies for each database and pathology.

Database	Pathology	Exact search string	Date of search
PubMed	Traumatic brain injury	("Acetylcysteine"[MeSH Terms] OR "N-acetylcysteine"[tiab] OR "N-acetyl-cysteine"[tiab] OR "N-acetyl cysteine"[tiab] OR "acetylcysteine"[tiab]) AND ("Brain Injuries, Traumatic"[MeSH Terms] OR "Brain Concussion"[MeSH Terms] OR "Head Injuries, Closed"[MeSH Terms] OR "Craniocerebral Trauma"[MeSH Terms] OR "traumatic brain injury"[tiab] OR "head injury"[tiab] OR "head trauma"[tiab] OR "brain trauma"[tiab] OR "concussion"[tiab] OR "postconcussion"[tiab] OR "post-concussion"[tiab] OR "mild TBI"[tiab] OR "mTBI"[tiab] OR "moderate TBI"[tiab] OR "severe TBI"[tiab] OR "craniocerebral trauma"[tiab] OR "closed head injury"[tiab]) AND (randomized controlled trial[pt] OR controlled clinical trial[pt] OR clinical trial[pt] OR "clinical trials as topic"[MeSH Terms] OR random*[tiab] OR trial[tiab] OR cohort[tiab] OR "cohort studies"[MeSH Terms] OR prospective[tiab] OR retrospective[tiab] OR "observational study"[pt] OR "open label"[tiab] OR "case series"[tiab]) AND ("1995/01/01"[PDAT] : "2025/12/31"[PDAT]) AND (humans[Filter])	10th of January 2026
PubMed	Alzheimer's disease	("Acetylcysteine"[MeSH Terms] OR "N-acetylcysteine"[tiab] OR "N-acetyl-cysteine"[tiab] OR "N-acetyl cysteine"[tiab] OR "acetylcysteine"[tiab]) AND ("Alzheimer Disease"[MeSH Terms] OR "Dementia"[MeSH Terms] OR "Cognitive Dysfunction"[MeSH Terms] OR alzheimer*[tiab] OR dementia[tiab] OR "cognitive impairment"[tiab] OR "cognitive	10th of January 2026

		decline"[tiab] OR "mild cognitive impairment"[tiab] OR "amnestic MCI"[tiab] OR "memory impairment"[tiab] OR "neurodegenerative dementia"[tiab]) AND (randomized controlled trial[pt] OR controlled clinical trial[pt] OR clinical trial[pt] OR "clinical trials as topic"[MeSH Terms] OR random*[tiab] OR trial[tiab] OR cohort[tiab] OR "cohort studies"[MeSH Terms] OR prospective[tiab] OR retrospective[tiab] OR "observational study"[pt] OR "open label"[tiab] OR "case series"[tiab]) AND ("1995/01/01"[PDAT] : "2025/12/31"[PDAT]) AND (humans[Filter])	
PubMed	Parkinson's disease	("Acetylcysteine"[MeSH Terms] OR "N-acetylcysteine"[tiab] OR "N-acetyl-cysteine"[tiab] OR "N-acetyl cysteine"[tiab] OR "acetylcysteine"[tiab]) AND ("Parkinson Disease"[MeSH Terms] OR "Parkinsonian Disorders"[MeSH Terms] OR parkinson*[tiab] OR parkinsonian[tiab] OR "substantia nigra"[tiab] OR "dopaminergic degeneration"[tiab] OR "idiopathic parkinsonism"[tiab]) AND (randomized controlled trial[pt] OR controlled clinical trial[pt] OR clinical trial[pt] OR "clinical trials as topic"[MeSH Terms] OR random*[tiab] OR trial[tiab] OR cohort[tiab] OR "cohort studies"[MeSH Terms] OR prospective[tiab] OR retrospective[tiab] OR "observational study"[pt] OR "open label"[tiab] OR "case series"[tiab]) AND ("1995/01/01"[PDAT] : "2025/12/31"[PDAT]) AND (humans[Filter])	10th of January 2026
PubMed	Multiple sclerosis	("Acetylcysteine"[MeSH Terms] OR "N-acetylcysteine"[tiab] OR "N-acetyl-cysteine"[tiab] OR "N-acetyl cysteine"[tiab] OR "acetylcysteine"[tiab]) AND ("Multiple Sclerosis"[MeSH Terms] OR "Multiple Sclerosis, Relapsing-Remitting"[MeSH Terms] OR "Multiple Sclerosis, Chronic	10th of January 2026

		Progressive"[MeSH Terms] OR "Demyelinating Diseases"[MeSH Terms] OR "multiple sclerosis"[tiab] OR "relapsing remitting"[tiab] OR "RRMS"[tiab] OR "secondary progressive"[tiab] OR "SPMS"[tiab] OR "primary progressive"[tiab] OR "PPMS"[tiab] OR "clinically isolated syndrome"[tiab] OR demyelinat*[tiab] OR "neuromyelitis optica"[tiab] OR "optic neuritis"[tiab]) AND (randomized controlled trial[pt] OR controlled clinical trial[pt] OR clinical trial[pt] OR "clinical trials as topic"[MeSH Terms] OR random*[tiab] OR trial[tiab] OR cohort[tiab] OR "cohort studies"[MeSH Terms] OR prospective[tiab] OR retrospective[tiab] OR "observational study"[pt] OR "open label"[tiab] OR "case series"[tiab]) AND ("1995/01/01"[PDAT] : "2025/12/31"[PDAT]) AND (humans[Filter])	
PubMed	Amyotrophic lateral sclerosis	("Acetylcysteine"[MeSH Terms] OR "N-acetylcysteine"[tiab] OR "N-acetyl-cysteine"[tiab] OR "N-acetyl cysteine"[tiab] OR "acetylcysteine"[tiab]) AND ("Amyotrophic Lateral Sclerosis"[MeSH Terms] OR "Motor Neuron Disease"[MeSH Terms] OR "amyotrophic lateral sclerosis"[tiab] OR "motor neuron disease"[tiab] OR "motor neurone disease"[tiab]) AND (randomized controlled trial[pt] OR controlled clinical trial[pt] OR clinical trial[pt] OR "clinical trials as topic"[MeSH Terms] OR random*[tiab] OR trial[tiab] OR cohort[tiab] OR "cohort studies"[MeSH Terms] OR prospective[tiab] OR retrospective[tiab] OR "observational study"[pt] OR "open label"[tiab] OR "case series"[tiab]) AND ("1995/01/01"[PDAT] : "2025/12/31"[PDAT]) AND (humans[Filter])	10th of January 2026
PubMed	Migraine	("Acetylcysteine"[MeSH Terms] OR "N-acetylcysteine"[tiab] OR "N-acetyl-cysteine"[tiab] OR "N-acetyl	10th of January 2026

		cysteine"[tiab] OR "acetylcysteine"[tiab] OR "NAC"[tiab]) AND ("Migraine Disorders"[MeSH Terms] OR migraine*[tiab] OR headache[tiab]) AND ("1995/01/01"[PDAT] : "2025/12/31"[PDAT]) AND (humans[Filter])	
PubMed	Epilepsy	Pubmed: ("Acetylcysteine"[MeSH Terms] OR "N-acetylcysteine"[tiab] OR "N-acetyl-cysteine"[tiab] OR "N-acetyl cysteine"[tiab] OR "acetylcysteine"[tiab]) AND ("Epilepsy"[MeSH Terms] OR "Seizures"[MeSH Terms] OR "Drug Resistant Epilepsy"[MeSH Terms] OR "Status Epilepticus"[MeSH Terms] OR epilep*[tiab] OR seizure*[tiab] OR convulsion*[tiab] OR "infantile spasm*"[tiab] OR "West syndrome"[tiab] OR "Lennox-Gastaut"[tiab] OR "Dravet syndrome"[tiab] OR "temporal lobe epilepsy"[tiab] OR "refractory epilepsy"[tiab] OR "absence seizure*"[tiab] OR "tonic-clonic"[tiab] OR "myoclonic"[tiab] OR "focal epilepsy"[tiab] OR "generalized epilepsy"[tiab]) AND (randomized controlled trial[pt] OR controlled clinical trial[pt] OR clinical trial[pt] OR "clinical trials as topic"[MeSH Terms] OR random*[tiab] OR trial[tiab] OR cohort[tiab] OR "cohort studies"[MeSH Terms] OR prospective[tiab] OR retrospective[tiab] OR "observational study"[pt] OR "open label"[tiab] OR "case series"[tiab]) AND ("1995/01/01"[PDAT] : "2025/12/31"[PDAT]) AND (humans[Filter])	11th of January 2026
Cochrane Library	Traumatic brain injury	#1 MeSH descriptor: [Acetylcysteine] explode all trees #2 ("N-acetylcysteine" OR "N-acetyl-cysteine" OR "N-acetyl cysteine" OR "acetylcysteine"):ti,ab,kw #3 #1 OR #2 #4 MeSH descriptor: [Brain Injuries, Traumatic] explode all trees #5 MeSH descriptor: [Brain Concussion] explode all trees #6 MeSH descriptor: [Head Injuries, Closed] explode all trees #7 MeSH descriptor: [Craniocerebral	13th of January 2026

		Trauma] explode all trees #8 ("traumatic brain injury" OR "head injury" OR "head trauma" OR "brain trauma" OR concussion OR postconcussion OR "post-concussion" OR "mild TBI" OR mTBI OR "moderate TBI" OR "severe TBI" OR "craniocerebral trauma" OR "closed head injury"):ti,ab,kw #9 #4 OR #5 OR #6 OR #7 OR #8 #10 #3 AND #9 with Publication Year from 1995 to 2025, in Trials	
Cochrane Library	Alzheimer's disease	#1 MeSH descriptor: [Acetylcysteine] explode all trees #2 ("N-acetylcysteine" OR "N-acetyl-cysteine" OR "N-acetyl cysteine" OR "acetylcysteine"):ti,ab,kw #3 #1 OR #2 #4 MeSH descriptor: [Alzheimer Disease] explode all trees #5 MeSH descriptor: [Dementia] explode all trees #6 MeSH descriptor: [Cognitive Dysfunction] explode all trees #7 (alzheimer* OR dementia OR "cognitive impairment" OR "cognitive decline" OR "mild cognitive impairment" OR "amnestic MCI" OR "memory impairment"):ti,ab,kw #8 #4 OR #5 OR #6 OR #7 #9 #3 AND #8 with Publication Year from 1995 to 2025, in Trials	13th of January 2026
Cochrane Library	Parkinson's disease	#1 MeSH descriptor: [Acetylcysteine] explode all trees #2 ("N-acetylcysteine" OR "N-acetyl-cysteine" OR "N-acetyl cysteine" OR "acetylcysteine"):ti,ab,kw #3 #1 OR #2 #4 MeSH descriptor: [Parkinson Disease] explode all trees #5 MeSH descriptor: [Parkinsonian Disorders] explode all trees #6 (parkinson* OR parkinsonian OR "substantia nigra" OR "dopaminergic degeneration" OR "idiopathic parkinsonism"):ti,ab,kw #7 #4 OR #5 OR #6 #8 #3 AND #7 with Publication Year from 1995 to 2025, in Trials	13th of January 2026
Cochrane Library	Multiple sclerosis	#1 MeSH descriptor: [Acetylcysteine] explode all trees #2 ("N-acetylcysteine" OR "N-acetyl-cysteine" OR "N-acetyl cysteine" OR "acetylcysteine"):ti,ab,kw #3 #1 OR #2 #4 MeSH descriptor: [Multiple Sclerosis] explode all trees #5 MeSH descriptor: [Multiple Sclerosis,	13th of January 2026

		Relapsing-Remitting] explode all trees #6 MeSH descriptor: [Multiple Sclerosis, Chronic Progressive] explode all trees #7 MeSH descriptor: [Demyelinating Diseases] explode all trees #8 ("multiple sclerosis" OR "relapsing remitting" OR RRMS OR "secondary progressive" OR SPMS OR "primary progressive" OR PPMS OR "clinically isolated syndrome" OR demyelinat* OR "neuromyelitis optica" OR "optic neuritis"):ti,ab,kw #9 #4 OR #5 OR #6 OR #7 OR #8 #10 #3 AND #9 with Publication Year from 1995 to 2025, in Trials	
Cochrane Library	Amyotrophic lateral sclerosis	#1 MeSH descriptor: [Acetylcysteine] explode all trees #2 ("N-acetylcysteine" OR "N-acetyl-cysteine" OR "N-acetyl cysteine" OR "acetylcysteine"):ti,ab,kw #3 #1 OR #2 #4 MeSH descriptor: [Amyotrophic Lateral Sclerosis] explode all trees #5 MeSH descriptor: [Motor Neuron Disease] explode all trees #6 ("amyotrophic lateral sclerosis" OR "motor neuron disease" OR "motor neurone disease"):ti,ab,kw #7 #4 OR #5 OR #6 #8 #3 AND #7 with Publication Year from 1995 to 2025, in Trials	14th of January 2026
Cochrane Library	Migraine	#1 MeSH descriptor: [Acetylcysteine] explode all trees #2 ("N-acetylcysteine" OR "N-acetyl-cysteine" OR "N-acetyl cysteine" OR "acetylcysteine"):ti,ab,kw #3 #1 OR #2 #4 MeSH descriptor: [Migraine Disorders] explode all trees #5 MeSH descriptor: [Migraine with Aura] explode all trees #6 MeSH descriptor: [Migraine without Aura] explode all trees #7 MeSH descriptor: [Headache Disorders] explode all trees #8 (migraine* OR "chronic migraine" OR "episodic migraine" OR cephalgia OR "headache disorder"):ti,ab,kw #9 #4 OR #5 OR #6 OR #7 OR #8 #10 #3 AND #9 with Publication Year from 1995 to 2025, in Trials	14th of January 2026
Cochrane Library	Epilepsy	#1 MeSH descriptor: [Acetylcysteine] explode all trees #2 ("N-acetylcysteine"	14th of January 2026

	OR "N-acetyl-cysteine" OR "N-acetyl cysteine" OR "acetylcysteine"):ti,ab,kw #3 #1 OR #2 #4 MeSH descriptor: [Epilepsy] explode all trees #5 MeSH descriptor: [Seizures] explode all trees #6 MeSH descriptor: [Drug Resistant Epilepsy] explode all trees #7 MeSH descriptor: [Status Epilepticus] explode all trees #8 (epilep* OR seizure* OR convulsion* OR (infantile NEXT spasm*) OR "West syndrome" OR "Lennox-Gastaut" OR "Dravet syndrome" OR (absence NEXT seizure*) OR "tonic-clonic" OR myoclonic OR "focal epilepsy" OR "generalized epilepsy"):ti,ab,kw #9 #4 OR #5 OR #6 OR #7 OR #8 #10 #3 AND #9 with Publication Year from 1995 to 2025, in Trials
