# Supplementary material for: N-Acetylcysteine in Neurological Disorders: A Systematic Review of Clinical and Translational Evidence Across Seven Disorders
Source: Int J Mol Sci. 2026 Mar 27;27(7):3076. doi: 10.3390/ijms27073076 (PMC13074174; doi:10.3390/ijms27073076)
Supplement: Supplementary file 1 [file ijms-27-03076-s001.zip › Supplementary material S4.pdf]

Table S2. Summary of oxidative stress and mechanistic biomarker data across included studies

Study	Condition	Biomarkers measured	Direction of effect
Gouda et al. [9]	TBI	MDA, IL-6, NSE, S100B	All significantly decreased in NAC group
Hagos et al. [12]	TBI	CSF metabolomics (7 GSH-centered pathways)	Enriched in NAC group
Adair et al. [14]	AD	SOD, GPx, GSH, TBARS (peripheral)	No significant differences
Holmay et al. [21]	PD	Brain GSH (7T MRS), blood GSH/GSSG	Brain GSH +55% (IV); blood GSH/GSSG increased
Coles et al. [22]	PD	Brain GSH (3T/7T MRS), catalase, GSH/GSSG, MDA, 4-HNE	Brain GSH: NS; catalase and GSH/GSSG: significantly increased; MDA, 4-HNE: NS
Schipper et al. [24]	MS	GSSG/GSH ratio, protein carbonyls, 8-epiPGF2α, 8-OHdG, tocopherols, carotenoids	GSSG/GSH decreasing trend (NS); all others: NS
Monti et al. [25]**	MS	FDG PET cerebral glucose metabolism	Significantly increased in select brain regions
Krysko et al. [26]	MS	Brain GSH (7T MRS)	NS (trend when measured closer to dose)
Khalatbari Mohseni et al. [27]	MS	MDA, NO, erythrocyte GSH	MDA significantly decreased; NO and GSH: NS

* *GSH = glutathione; GSSG = oxidized glutathione; MDA = malondialdehyde; IL-6 = interleukin-6; NSE = neuron-specific enolase; SOD = superoxide dismutase; GPx = glutathione peroxidase; TBARS = thiobarbituric acid reactive substances; 4-HNE = 4-hydroxynonenal; NO = nitric oxide; 8-OHdG = 8-hydroxy-2-deoxyguanosine; MRS = magnetic resonance spectroscopy; NS = not significant; IV = intravenous.*

***FDG PET cerebral glucose metabolism was included as a mechanistic biomarker; it is not classified as an oxidative stress biomarker in the main text count (8 of 23 studies).*
