# Supplementary material for: N-Acetylcysteine in Neurological Disorders: A Systematic Review of Clinical and Translational Evidence Across Seven Disorders
Source: Int J Mol Sci. 2026 Mar 27;27(7):3076. doi: 10.3390/ijms27073076 (PMC13074174; doi:10.3390/ijms27073076)
Supplement: Supplementary file 1 [file ijms-27-03076-s001.zip › Supplementary material S5.pdf]

Table S3: Summary of adverse events across included studies

**Study**	Condition	NAC dose/route	Adverse events reported	Discontinuations due to AEs
Hoffer et al. [8]	TBI	4g/day → 3g/day oral	None reported	None
Gouda et al. [9]	TBI	150mg/kg load + 50mg/kg/4h oral/enteral	None attributable to NAC	None
Clark et al. [11]	TBI	Oral + probenecid	None in treatment group	None (1 placebo withdrew — rash)
Hagos et al. [12]	TBI	ORAL + probenecid	AEs reported in Clark et al. [11]	None
Vedaei et al. [10]	TBI	IV 50mg/kg/wk + oral 1000mg/day	Well tolerated, no major AEs	None
Amen et al. [13]	TBI	NAC in multicomponent supplement	No significant AEs	None
Adair et al. [14]	AD	50mg/kg/day oral	Fatigue (26%), headache (26%), appetite change (22%), disposition change (22%); NS vs placebo	None
Chan et al. [15]	AD	NF (NAC 600mg)	No serious AEs	None
Remington et al. [16]	AD	NF (NAC 600mg)	No serious AEs	None
Remington et al. [17]	AD	NF (NAC 600mg)	No serious AEs	None
Remington et al. [18]	AD	NF (NAC 600mg)	No serious AEs	None
Monti et al. [19]	PD	IV 50mg/kg/wk + oral 600mg x2/day	No significant AEs	None
Monti et al. [20]	PD	IV 50mg/kg/wk + oral 500mg x2/day	No significant AEs	None
Holmay et al. [21]	PD	IV 150mg/kg single dose	Not reported	None
Coles et al. [22]	PD	6000mg/day oral	Abdominal pain (n=1), constipation (n=1)	Resolved after discontinuation

Table S3: Summary of adverse events across included studies

**Study**	Condition	NAC dose/route	Adverse events reported	Discontinuations due to AEs
Yulug et al. [23]	PD	CMA (NAC 2.55g/dose) x2/day	No significant difference vs placebo	None
Schipper et al. [24]	MS	5g/day oral + GA	Headache (n=3), abdominal distension (n=3), asthenia (n=2)	None
Monti et al. [25]	MS	IV 50mg/kg/wk + oral 500mg x2/day	No specific AEs reported	None
Krysko et al. [26]	MS	1250mg TID oral	≥1 AE in 60% NAC vs 80% placebo (NS)	None
Khalatbari Mohseni et al. [27]	MS	600mg x2/day oral	Shortness of breath (n=1, resolved on discontinuation)	1 patient
Louwerse et al. [28]	ALS	50mg/kg/day subcutaneous	Rash, pain at injection site	3 patients (rash/injection site pain)
Vyth et al. [29]	ALS	NAC SC + other antioxidants	Local injection-site reactions, 3 abscesses	Not specified
Visser et al. [30]	Migraine	NEC (NAC 600mg + Vit E + Vit C) x2/day	None reported	None

Legend: *AE = adverse event; NS = not significant; NF = nutraceutical formulation; CMA = combined metabolic activators; NEC = NAC + vitamin E + vitamin C; GA = glatiramer acetate; SC = subcutaneous; IV = intravenous; TID = three times daily.*
